# Association between proximity to a lead‐releasing facility and cognition in diverse cohorts

**DOI:** 10.1002/alz.71446

**Published:** 2026-05-01

**Authors:** Scarlet Cockell, Kelly M. Bakulski, Ai‐Lin Tsai, Yike Liu, Amanda J. Goodrich, Chinomnso N. Okorie, Stacey Alexeeff, Rachel A. Whitmer, Paola Gilsanz, Kathryn C. Conlon

**Affiliations:** ^1^ Department of Epidemiology School of Public Health University of Michigan Ann Arbor Michigan USA; ^2^ Division of Research Kaiser Permanente Northern California Pleasanton California USA; ^3^ Department of Public Health Sciences, School of Medicine University of California Davis Davis California USA

**Keywords:** cognition, cohort, dementia, environmental exposures, epidemiology, industrial pollution, lead

## Abstract

**BACKGROUND:**

The associations between adult lead exposure and late‐life cognition are largely unknown.

**METHODS:**

In two cohorts, Kaiser Healthy Aging and Diverse Life Experiences (KHANDLE, *n* = 1638) and Study of Healthy Aging in African Americans (STAR, *n* = 741), we assessed residential proximity to lead‐releasing facilities for association with domain‐specific cognition 2 years later. Linear regression models adjusted for age, sex, race/ethnicity, income, education, marital status, smoking status, and alcohol consumption. We meta‐analyzed across cohorts.

**RESULTS:**

Average age was 76.1 (KHANDLE), 68.8 years (STAR); average residential distance to a lead facility was 8.2 km (KHANDLE), 3.6 km (STAR). In meta‐analysis, for every 5 km closer a residence was located to a lead‐releasing facility, episodic memory scores 2 years later were −0.05 (95% confidence interval: −0.08, −0.02) standard deviation lower.

**DISCUSSION:**

Residential proximity to a lead‐releasing facility was associated with poorer cognition 2 years later among adults in two cohorts.

## Highlights

- Proximity to lead facilities was associated with lower average cognition scores.
- Lead exposure impacted domain‐specific cognition over relatively short periods of time.
- Lead and lower cognition associations were also observed among higher education strata.
- Lead exposure in adulthood is an environmental health concern.

## 1 BACKGROUND

Cognitive impairment and decline in later life are increasingly urgent public health concerns; particularly as today's older adults are living longer.^1^ Impairment in cognitive domains such as episodic memory, semantic memory, and executive function can significantly compromise independence and overall quality of life, contributing to elevated risks of physical disability and hospitalization.^2^ Even subtle declines in cognitive performance strongly predict the subsequent onset of dementia.^3^ In the United States, nearly two thirds of adults experience some level of cognitive impairment by age 70.^4^ Dementia, and other related subtypes, rank among the most expensive chronic health conditions in the United States, primarily due to the extended disease duration and the intensive care required.^4^, ^5^ The annual estimated cost of dementia care per individual in 2025 for the United States, including medical and long‐term care, was $231.7 billion.^6^ With disease‐modifying treatments still limited, identifying modifiable risk factors is a critical strategy for preventing the onset and progression of dementia.^6^

Environmental toxicants such as air pollution have been identified by the Lancet Commission as a modifiable risk factor for cognitive aging.^6^, ^7^ Building on the strong foundation of existing air pollution and dementia research by investigating sources that contribute to air pollution, such as lead‐releasing facilities, we may provide opportunities for intervention.^6^ Another important component of the chemical exposome is lead (i.e., Pb). Historically, lead was widely used in gasoline, household paints, tobacco products, water systems, and industrial processes, resulting in extensive environmental contamination and widespread population exposure.^8^, ^9^ Although regulatory measures have reduced average lead levels, legacy contamination persists due to aging infrastructure, industrial emissions, and soil contamination near smelting or recycling facilities.^1^, ^3^, ^9^ Additionally, lead exposure from residentially located lead‐releasing industrial facilities is still common in the United States. In fact, Toxics Release Inventory (TRI) facilities releasing lead have been associated with blood lead levels in nearby community settings and health outcomes.^10^, ^11^, ^12^, ^13^, ^14^, ^15^, ^16^ In 2023 there were 7507 lead‐releasing facilities in the United States, and 425 lead‐releasing facilities in California.^17^ Many of these facilities are located within significantly populated areas, with some facilities located in counties of > 100,000 people.^17^ Substantial evidence has linked early life and occupational lead exposures to adverse neurocognitive outcomes, including reduced intellectual functioning and long‐term cognitive deficits.^1^, ^18^ The long biological half‐life (25–30 years) of lead and preferential accumulation in bone tissue can result in sustained internal exposure that may continue to affect health years after initial contact.^19^, ^20^ Emerging studies suggest that cumulative lead exposure across the life course, even at low levels, may affect memory, attention, and executive function.^21^, ^22^ However, results are inconsistent and data remain sparse.^9^, ^23^

Little is known about how spatially patterned industrial exposures, such as residential proximity to lead‐emitting facilities, relate to cognitive aging in diverse populations. This limitation constrains the ability to evaluate long‐term effects of environmental lead exposure in community‐based settings, especially in the context of environmental health disparities. Communities with higher proportions of historically racially marginalized populations often experience disproportionate exposure to lead.^24^, ^25^, ^26^ However, comparatively less attention has been given to evaluating these disparities in lead exposure among adult populations. Few US‐based studies have evaluated associations between spatial indicators of lead exposure and cognitive outcomes while incorporating lag periods. This is particularly salient in California, where legacy industrial activity and demographic diversity create unique environmental and social exposure contexts. This study addresses these gaps, and builds on the foundation of air pollution and dementia research, by examining whether residential proximity to lead‐releasing facilities is associated with cognitive performance among older adults in California.

**RESEARCH IN CONTEXT**

1. **Systematic review**: Current and past literature was reviewed through traditional sources (Google Scholar, PubMed). Few publications explored the relationship between adult lead exposure and cognitive aging in diverse populations.
2. **Interpretation**: In two large, California‐based cohorts of older adults, we showed that residential proximity to an industrial lead‐releasing facility was associated with lower average episodic memory, semantic memory, executive function, and global cognition, measured 2 years later. Lead exposure in adulthood may be a modifiable risk factor for cognitive impairment, and efforts to reduce exposures may contribute to prevention. This analysis contributes to the growing knowledge of the exposome by incorporating the physical environment.
3. **Future directions**: Future research exploring additional exposomic components, including chemical mixtures, quantities of lead released, with repeated chemical and cognitive measures are needed to elucidate how lead exposure in adulthood impacts cognitive decline.

Given the limited number of studies, and inconsistent results on industrial lead exposure and cognition in adulthood, we were motivated to perform a geospatial analysis of residential lead exposure across multiple cognitive domains. In this study, we examined whether residential proximity to lead‐releasing facilities was associated with verbal episodic memory, semantic memory, executive function, and global cognitive performance, measured 2 years later among a diverse group of older adults in California.

## 2 METHODS

### 2.1 Study participants

This analysis included two mutually exclusive longitudinal cohort studies on aging among older adults in Northern California: the Kaiser Healthy Aging and Diverse Life Experiences (KHANDLE) and the Study of Healthy Aging in African Americans (STAR). The institutional review board at Kaiser Permanente Northern California approved both cohort studies (1278966, 1279068). All participants provided written informed consent.

The KHANDLE cohort included adults ages ≥ 65 years living in the Sacramento and San Francisco Bay communities of California, USA. To recruit a diverse and representative cohort, stratified random sampling by race, ethnicity, and education was performed.^27^ Participants were eligible for KHANDLE if they were members of Kaiser Permanente Northern California, an integrated health‐care delivery system, and completed at least one voluntary health exam called the Multiphasic Health Checkup (MHC) between the years 1964 and 1985, spoke English or Spanish, and were ≥ 65 years as of January 1, 2017.^28^, ^29^ Participants were excluded if they had a recorded diagnosis of dementia, chronic obstructive pulmonary disease, congestive heart failure hospitalizations, end‐stage renal disease, or dialysis.^29^, ^30^, ^31^ The KHANDLE study is ongoing, and these analyses include information collected at study enrollment, which occurred between April 2017 and December 2018. Further details on KHANDLE cohort design are available at Peterson et al.^27^ and Hernandez Saucedo et al.^29^ Participants were excluded from the analytic sample if they were missing any cognitive assessment measure (*n* = 25). To protect participant anonymity due to small sample size, those identifying as Native American were excluded from this analysis.

The STAR cohort included Black adults ages ≥ 50 years with residential addresses in the San Francisco Bay region of California, USA. Cohort recruitment was performed using stratified random sampling by age and education attainment.^32^ Participants were eligible for STAR if they were members of Kaiser Permanente Northern California, had at least one MHC visit between 1964 and 1985, identified as African American or Black, and were age ≥ 50 on January 1, 2018. Cohort comorbidity exclusion criteria is the same as KHANDLE. The STAR study is ongoing, and these analyses include information collected at study enrollment, which took place between November 2017 and March 2020. Further details on STAR cohort design are available at Whitmer et al.^32^ Participants were excluded from the analytic sample if they were missing any cognitive assessment measure (*n* = 5). To protect participant anonymity due to small sample size, those identifying as Native American were excluded from this analysis.

### 2.2 Study design

**FIGURE 1:**
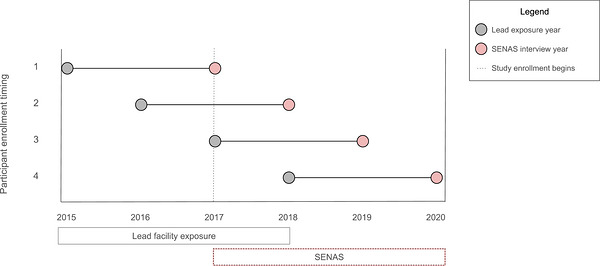
Study design timing: Each participant had a single lead exposure measure (gray) and 2 years later, had a single cognitive measure (red). The year of each participant's measures depended on the timing of their cognitive testing, performed using the Spanish and English Neuropsychological Assessment Scales (SENAS). SENAS for all study participants were completed once during enrollment between 2017 and 2020. Thus, the exposure measures generated from residential address proximity to lead‐releasing facilities were linked between 2015 and 2018. All linkages follow a 2‐year lag design during which exposures were measured before cognitive outcomes.

In this analysis, each participant had a single exposure measure and a single cognitive outcome measure that were separated by 2 years in a lagged design (Figure 1). Based on the year of a given participant's cognitive assessment (study range: 2017–2020), we counted backward 2 years for that participant's exposure measure (study range: 2015–2018). For example, if a participant had a cognitive test in 2020, we linked their exposure measure from 2018 for analysis. Because pathological changes occur within the brain before clinical changes are observed, we elected to use a 2‐year lag window to maximize our sample size and minimize our exposure measurement error.^33^

### 2.3 Exposure measures

The US Environmental Protection Agency (EPA) maintains the TRI. The TRI, established in 1986, provides open access to nationwide toxic chemical release data reported yearly by industrial and federal facilities across the United States.^34^ The TRI Toxics Tracker stores data from the past 10 years, with detailed information on geography, facility, industry sector, chemical, and release type.^17^ The TRI reports pounds of emissions released from TRI facilities in the following categories: total, air, water, land, off‐site, and waste managed. Chemical and facility data for the entire state of California from 2012 to 2021 were extracted from the TRI Toxics Tracker. To ensure our exposure occurred before our outcome measures, we first restricted the TRI exposure data to the years 2015 to 2018 (*n* = 544 facilities). Next, we restricted to facilities releasing lead (designated as either “lead compounds” or “lead”; *n* = 525 facilities), then to facilities that released > 0 pounds of lead per year (*n* = 453 facilities).

Residential addresses at ages 40 and 65 were self‐reported to each of the cohorts, and we selected the residential address at the age nearest to the study enrollment age for linkage. Participant addresses were geocoded into coordinates of latitude and longitude using ArcGIS (average match score: 98.9 KHANDLE, 97.5 STAR). A portion (about 10%) of participants in both cohorts provided only their city‐level residential information, but not a street‐level address. In these cases, participants were geocoded as the latitude and longitude of the centroid of the city. Geocoded residential addresses were linked to the nearest lead‐releasing TRI facility, 2 years prior to cognitive testing. We created four distance exposure measures that were used in this analysis. We calculated the distance in kilometers from a participant's residence to the TRI facility, which we used as our continuous exposure variable. To examine buffer sizes around the facilities, we then created three binary variables for living (yes, no) within each of three distances (1.5 km, 3 km, or 5 km) around a lead‐releasing TRI facility. The reference group for each categorical distance measure was “no.” We used both continuous and categorical (1.5 km, 3 km, 5 km) exposure measures for statistical analysis.

### 2.4 Cognitive measures

Cognitive outcomes of verbal episodic memory, semantic memory, executive function, and global cognition were derived from Spanish and English Neuropsychological Assessment Scales (SENAS).^35^ SENAS methodology has been previously described.^35^ Briefly, SENAS was developed with item response theory methodology to allow for valid comparisons of cognition and cognitive change across diverse groups.^35^ The episodic memory domain was a composite measure of item response, category fluency, working memory, learning trials, and delayed free recall.^36^ Semantic memory domain was a composite of object naming and picture association.^36^ The executive function domain was a composite measure of category fluency, phonemic fluency, and working memory.^36^ Finally, the global cognition domain was an average of the episodic memory, semantic memory, and executive function scores.^35^, ^36^, ^37^ Descriptions of test administration, development, and guidelines have been described previously.^35^ Each participant completed the SENAS examination once, in either English or Spanish, during baseline interviews between 2017 and 2020. During 2017 to 2019, in‐person interviews were conducted by study personnel. In 2020, interviews were conducted via telephone, due to the COVID‐19 pandemic.

Each cognitive outcome was *z* score standardized, separately for KHANDLE and STAR, to allow for more meaningful interpretation and comparability across participants. To generally understand cognitive trends within each exposure buffer, we dichotomized each cognitive domain score into high (> 0) and low (≤ 0) for descriptive analyses. Continuous cognitive domain scores were used in statistical analysis.

### 2.5 Covariate measures

Demographic and health information, including age, sex (male, female), race and ethnicity (Asian, Black, LatinX, Native American, White, or other racial/ethnic identity), education (less than or equal to high school, greater than high school), marital status (married/living as married, not married), smoking status (never, former, current), and alcohol consumption (never, less than once a week, 1–6 days per week, every day), were collected via in‐person interview at enrollment. Average household census tract income was acquired from the US Census Bureau database for years 2015 to 2017, and linked to geocoded residential addresses. For the small number of people who completed study interviews in 2020, we used 2017 average income data for household census tracts, as 2018 data were unavailable.

### 2.6 Statistical analysis

We performed a single imputation for those missing covariate information on education (*n* = 1, 0.06% KHANDLE; *n* = 3, 0.4% STAR), marital status (*n* = 26, 1.5% KHANDLE; *n* = 17, 2.2% STAR), average census tract income (*n* = 3, 0.1% KHANDLE), smoking status (*n* = 6; 0.4% KHANDLE), and alcohol consumption (*n* = 12; 0.7% KHANDLE) using the fully conditional specification method.^38^ For KHANDLE, race/ethnicity was included as a covariate to inform the imputation. Descriptive statistics were calculated on the included and excluded samples using mean and standard deviation for continuous variables, and frequency and percent for categorical variables. Because KHANDLE and STAR have different geographic, age, and race/ethnicity recruitment strategies, we performed all analyses separately in each cohort. We calculated bivariate sample characteristics by exposure distance (within 1.5 km, 3 km, and 5 km) and high/low values for each cognitive domain (episodic memory, semantic memory, executive function, global cognition), and compared the distributions using a Satterthwaite *t* test for continuous variables and a Fisher exact test for categorical variables.

Our primary analyses tested the adjusted associations between residential distance to a lead‐releasing facility and cognitive score 2 years later using multivariable linear regression. We evaluated distance to the nearest lead‐releasing facility as a continuous measure as well as categorically (buffers with radii of 1.5 km, 3 km, 5 km). We examined each distance measure for association with each cognitive domain score, adjusting for age at cognitive testing and sex (minimally adjusted), and further adjusted for race/ethnicity, average census tract income, education, marital status, smoking status, and alcohol consumption (fully adjusted). For comparison, we also report the associations for a 1‐year increase in age from the fully adjusted models. Race and ethnicity was included in fully adjusted models for KHANDLE only; race and ethnicity was not adjusted for in STAR as all participants recruited identified as Black. We performed a conversion of the continuous distance measure, which was modeled as per 1 km increase, by multiplying effect estimates by −5. This allowed us to interpret our findings as per every 5 km closer to a lead‐releasing facility. To increase statistical power and generalizability, we meta‐analyzed results across the KHANDLE and STAR cohorts, using random‐effects models. For comparison, from these same models, we also reported the associations for a 1‐year increase in age.

### 2.7 Sensitivity analyses

We conducted multiple sensitivity analyses to interrogate the robustness of our results. We performed a complete case sensitivity analysis to assess how our findings are impacted by different assumptions of missing data. Participants with missing information for any aforementioned covariates were excluded, resulting in final analytic samples of *n* = 1590 for KHANDLE, and *n* = 721 for STAR. Effect modification by age (using categories of < 70, 70–74, 75–79, ≥ 80) and education (≤ high school, trade school or college, graduate school) were assessed in stratified analyses. To evaluate potential exposure misclassification, we conducted several analyses. First, we restricted analytic samples for both cohorts to include only participants who provided street‐level residential address information (KHANDLE *n* = 1477; STAR *n* = 687). Second, we applied a minimum annual release threshold of ≥ 100 pounds of lead for each facility and conducted analysis on this restricted sample.^34^ Third, we performed a stratified analysis by mode of release (air, water, land). Fourth, to address potential confounding by spatial features, we performed a mixed effects regression with a random intercept for census tract. Finally, in exploratory analyses we restricted our sample to facilities releasing ≥ 100 pounds of lead by air and conducted analysis on this sample for the KHANDLE cohort (analysis was not performed in STAR due to sample size).

### 2.8 Extended analyses

In extended analyses to address confounding by ambient air pollution, we included annual average fine particulate matter with a diameter ≤ 2.5 micrometers (PM_2.5_) as a covariate in our fully adjusted models. Individual‐level average PM_2.5_ exposures were computed by averaging daily estimates of PM_2.5_ for one calendar year 2 years before cognitive testing (consistent with the lead exposure measures), and linked to geocoded residential addresses. For example, if a cognitive assessment was conducted in 2020, PM_2.5_ exposure from 2018 was used. PM_2.5_ exposures at 1 km resolution were generated from a validated ensemble model (cross‐validated *R* ^2^ = 0.89 for 1‐year average PM_2.5_) which combines data from satellite measurements, meteorological variables, land‐use variables, elevation, and chemical transport model predictions via three machine learning algorithms, with further details described elsewhere.^39^ To gain insight into the relationship between annual PM_2.5_ and continuous distance to a lead TRI facility, we calculated Pearson correlation coefficients for KHANDLE and STAR.

We used SAS statistical software (version 9.4) and R statistical software (version 4.1) for our analyses. Code to produce the analyses is available at https://github.com/bakulskilab.

## 3 RESULTS

### 3.1 Lead‐releasing facilities descriptive statistics

**FIGURE 2:**
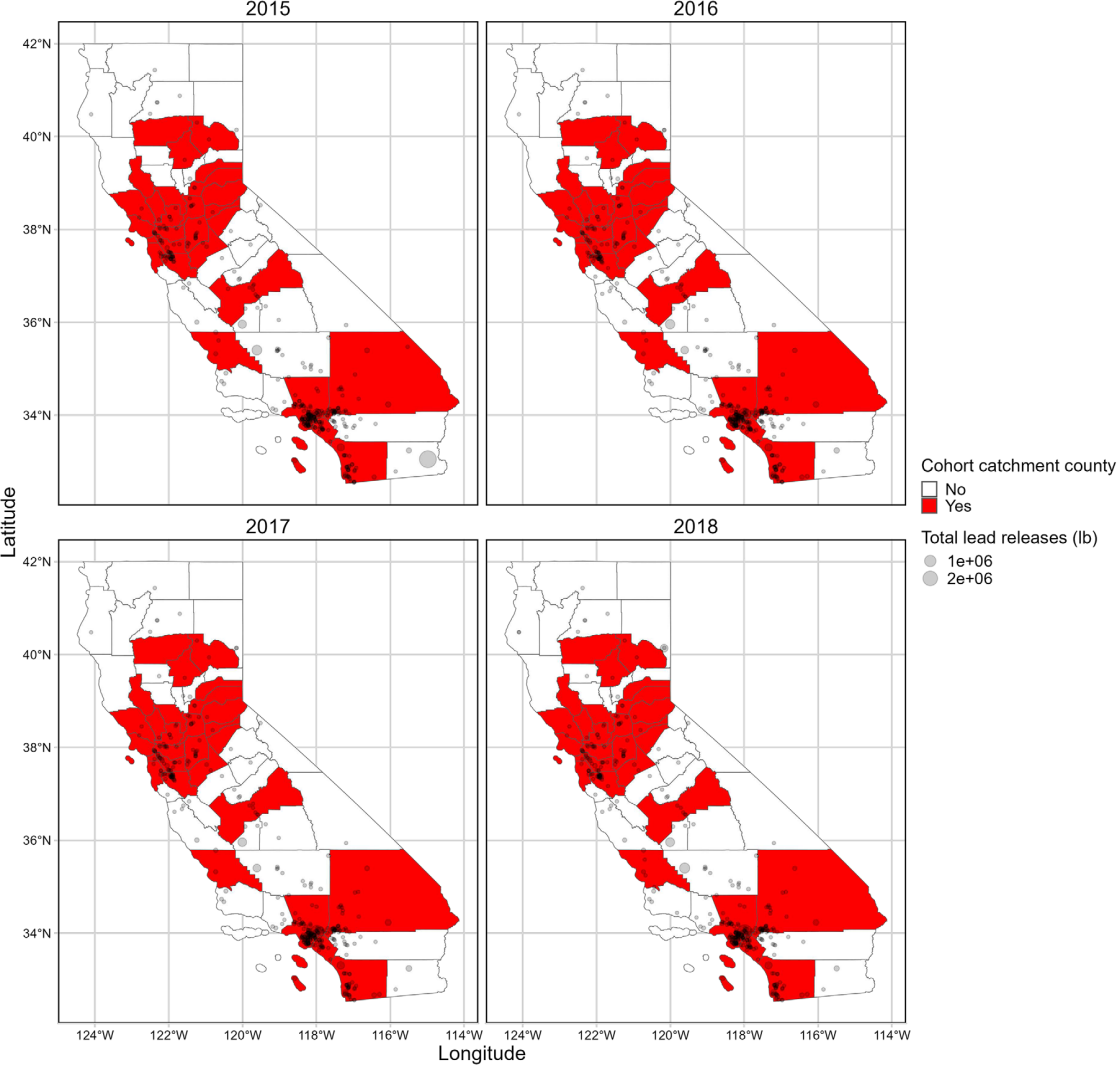
Distribution of Toxics Release Inventory (TRI) lead‐releasing facilities by cohort catchment counties in California, years 2015 to 2018. Points represent the TRI facility location, scaled by the total amount of lead released in pounds per year. Study participants resided across a region of 28 counties in California and are colored red. Counties outside the catchment area are depicted in white.

The number of lead‐releasing facilities per county fluctuated by year (Figure 2). Study participants resided across a region of 28 counties in California (Figure 2). Across all exposure years, Los Angeles consistently had the greatest number of TRI facilities per county, with 106 facilities in 2018. The amount of lead released varied by year, mode of release, county, and facility.

### 3.2 Study sample descriptive statistics

For the KHANDLE cohort, *n* = 1663 participants were eligible for this study. We excluded those without cognitive assessments (*n* = 25), leaving *n *= 1638 included in our analytic sample (Figure S1A in supporting information). Relative to the included sample, those excluded were older (mean 80 vs. 76 years), had greater than high school degree (72% vs. 83%), lived closer to a TRI facility (mean 7.4 km vs. 8.2 km), and were more likely to be Black (36% vs. 25%; Table S1 in supporting information). For the STAR cohort, *n* = 746 participants were eligible for this study. After excluding those without cognitive assessments (*n* = 5), our included analytic sample size was *n* = 741 (Figure S1B). Overall, participants in the STAR cohort lived closer to lead‐releasing facilities than those in the KHANDLE cohort (Figure S2 in supporting information). Relative to the included sample, those excluded were older (mean 69 vs. 68 years), had greater than high school degree (80% vs. 81.8%), and lived closer to a TRI facility (mean 3 km vs. 3.6 km; Table S1).

Among the KHANDLE included sample, the average age at cognitive assessment was 76 years, 58.9% were female, 25.8% identified as Black, 25% as Asian, 19.7% as LatinX, and 29.5% as White (Table 1). The average distance between residence and lead‐releasing facility was 8.21 km. Among the STAR included sample, the average age at cognitive assessment was 68 years, 68.6% were female, 98.7% identified as Black, and 0.8% LatinX (Table 2). The average distance between residence and lead‐releasing facility was 3.56 km. In KHANDLE, 60 (3.7%) participants lived within 1.5 km of a lead‐releasing facility, 326 (19.9%) lived within 3 km of a lead‐releasing facility, and 742 (45.3%) lived within 5 km. In STAR, 84 (11.3%) participants lived within 1.5 km of a lead‐releasing facility, 343 (46.3%) lived within 3 km of a lead‐releasing facility, and 620 (83.7%) lived within 5 km. Overall, those in the STAR cohort tended to live closer to lead‐releasing facilities, were more likely to be female, and were younger on average, than those in KHANDLE[Table alz71446-tbl-0001], [Table alz71446-tbl-0002].

**TABLE 1 alz71446-tbl-0001:** Kaiser Healthy Aging and Diverse Life Experiences (KHANDLE) analytic sample descriptive statistics by residential distance buffer around a lead‐releasing facility.

		Residential distance buffer								
		Live within 1.5 km lead facility			Live within 3 km lead facility			Live within 5 km lead facility		
Characteristic	Overall *N* = 1638	No *N* = 1578	Yes *N* = 60	*p* value^a^	No *N* = 1312	Yes *N* = 326	*p* value^a^	No *N* = 896	Yes *N* = 742	*p* value^a^
**Global cognition** ^b^	0.01 (0.81)	0.02 (0.81)	−0.25 (0.94)	0.03	0.06 (0.79)	−0.18 (0.86)	<0.01	0.10 (0.76)	−0.09 (0.86)	<0.01
**Executive function** ^b^	0.01 (1.0)	0.03 (0.99)	−0.35 (1.11)	0.01	0.06 (0.99)	−0.17 (1.02)	<0.01	0.06 (0.97)	−0.05 (1.02)	0.02
**Episodic memory** ^b^	0.01 (1.0)	0.01 (0.99)	−0.13 (1.16)	0.34	0.04 (0.99)	−0.13 (1.03)	<0.01	0.09 (0.99)	−0.09 (1.01)	<0.01
**Semantic memory** ^b^	0.02 (0.99)	0.03 (0.99)	−0.26 (1.03)	0.03	0.08 (0.97)	−0.22 (1.04)	<0.01	0.13 (0.92)	−0.13 (1.06)	<0.01
**Average distance to facility (km)**	8.2 (6.9)	8.5 (6.9)	1.1 (0.4)	<0.01	9.7 (6.9)	2.1 (0.6)	<0.01	12.4 (6.9)	3.2 (1.1)	<0.01
**Total lead releases (lbs)** ^c^	2313.6 (5344.3)	2328.8 (5381.6)	1914.5 (4265.8)	0.46	2034.4 (4645.0)	3437 (7432.8)	<0.01	1641.2 (4173.4)	3125.5 (6391.7)	<0.01
**Air lead releases (lbs)**	11.1 (29.8)	10.9 (29.1)	17.8 (43.3)	0.22	9.2 (26.2)	19.0 (40.2)	<0.01	5.6 (17.4)	17.8 (38.8)	<0.01
**Water lead releases (lbs)**	1.6 (7.2)	1.6 (7.2)	1.3 (7.5)	0.76	1.6 (7.4)	1.2 (6.6)	0.32	1.8 (7.9)	1.2 (6.3)	0.07
**Land lead releases (lbs)**	10.4 (232.5)	10.6 (236.8)	4.1 (30.7)	0.36	12.1 (259.4)	3.5 (27.3)	0.23	17.1 (313.6)	2.3 (21.9)	0.15
**PM_2.5_ (µg/m** ^c^)	7.5 (1.5)	7.4 (1.5)	7.8 (1.4)	0.01	7.4 (1.4)	7.6 (1.4)	<0.01	7.2 (1.5)	7.7 (1.3)	<0.01
**Age at interview**	76.1 (7.1)	76.1 (7.1)	74.9 (5.9)	0.13	76.1 (7.1)	75.8 (7.2)	0.43	76.3 (7.1)	75.8 (7.3)	
**Sex**				0.89			0.13			0.01
Male	674 (41.1%)	650 (41.2%)	24 (40.0%)		552 (42.1%)	122 (37.4%)		393 (43.9%)	281 (37.9%)	
Female	964 (58.9%)	928 (58.8%)	36 (60.0%)		760 (57.9%)	204 (62.6%)		503 (56.1%)	461 (62.1%)	
**Race/ethnicity**				0.81			<0.01			<0.01
Asian	410 (25.0%)	395 (25.0%)	15 (25.0%)		346 (26.4%)	64 (19.6%)		255 (28.5%)	155 (20.9%)	
Black	422 (25.8%)	406 (25.7%)	16 (26.7%)		304 (23.2%)	118 (36.2%)		154 (17.2%)	268 (36.1%)	
LatinX	322 (19.7%)	308 (19.5%)	14 (23.3%)		246 (18.8%)	76 (23.3%)		183 (20.4%)	139 (18.7%)	
White	484 (29.5%)	469 (29.7%)	15 (25.0%)		416 (31.7%)	68 (20.9%)		304 (33.9%)	180 (24.3%)	
**Education**				<0.01			<0.01			0.16
≤ High school	274 (16.7%)	255 (16.2%)	19 (31.7%)		202 (15.4%)	72 (22.1%)		139 (15.5%)	135 (18.2%)	
> High school	1364 (83.3%)	1323 (83.8%)	41 (68.3%)		1110 (84.6%)	254 (77.9%)		757 (84.5%)	607 (81.8%)	
**Marital status**				0.42			<0.01			<0.01
Married/ living as married	933 (57.0%)	902 (57.2%)	31 (51.7%)		785 (59.8%)	148 (45.4%)		553 (61.7%)	380 (51.2%)	
Not married	705 (43.0%)	676 (42.8%)	29 (48.3%)		527 (40.2%)	178 (54.6%)		343 (38.3%)	362 (48.8%)	
**Average census tract income**	121,338 (52,304)	122,237 (52,696)	97,694 (33,053)	<0.01	128,293 (54,558)	93,348 (28,127)	<0.01	134,094 (54,478)	105,934 (44,995)	<0.01
**Smoking status**				0.96			0.29			0.34
Never	912 (55.7%)	879 (55.7%)	33 (55.0%)		735 (56.0%)	177 (54.3%)		508 (56.7%)	404 (54.4%)	
Past	677 (41.3%)	651 (41.3%)	26 (43.3%)		534 (40.7%)	143 (43.9%)		358 (40.0%)	319 (43.0%)	
Current	49 (3.0%)	48 (3.0%)	1 (1.7%)		43 (3.3%)	6 (1.8%)		30 (3.3%)	19 (2.6%)	
**Alcohol consumption**				0.10			0.07			0.34
Never	481 (29.4%)	459 (29.1%)	22 (36.7%)		376 (28.7%)	105 (32.2%)		241 (26.9%)	240 (32.3%)	
Less than once a week	572 (34.9%)	549 (34.8%)	23 (38.3%)		447 (34.1%)	125 (38.3%)		309 (34.5%)	263 (35.4%)	
1–6 days per week	425 (25.9%)	417 (26.4%)	8 (13.3%)		355 (27.1%)	70 (21.5%)		250 (27.9%)	175 (23.6%)	
Every day	160 (9.8%)	153 (9.7%)	7 (11.7%)		134 (10.2%)	26 (8.0%)		96 (10.7%)	64 (8.6%)	

*Note*: Mean (SD) for continuous variables, *N* (%) categorical.

^a^ Fisher exact test, Satterthwaite *t* test.

^b^ Cognitive measures are *z* score standardized.

^c^ Total lead releases is a combination of air, land, water, and off‐site lead releases reported by the facility to the Toxics Release Inventory. Off‐site releases was not included in this analysis and therefore when combined, proportions of lead released through air, water, and land may not add to the total lead release number listed. Average census tract income rounded to the nearest whole number.

Abbreviations: km, kilometer; lbs, pounds; PM_2.5_, atmospheric particulate matter with a diameter < 2.5 micrometers; SD standard deviation.

**TABLE 2 alz71446-tbl-0002:** Study of Healthy Aging in African Americans (STAR) analytic sample descriptive statistics by residential distance buffer around a lead‐releasing facility.

		Residential distance buffer								
		Live within 1.5 km			Live within 3 km			Live within 5 km		
Characteristic	Overall *N* = 741	No *N* = 657	Yes *N* = 84	*p* value^a^	No *N* = 398	Yes *N* = 343	*p* value^a^	No *N* = 121	Yes *N* = 620	*p* value^a^
**Global cognition** ^b^	0.01 (0.81)	0.04 (0.80)	−0.18 (0.85)	0.03	0.1 (0.78)	−0.09 (0.82)	<0.01	0.23 (0.73)	−0.03 (0.82)	<0.01
**Executive function** ^b^	0.01 (0.99)	0.04 (0.98)	−0.16 (1.05)	0.10	0.12 (0.95)	−0.11 (1.02)	<0.01	0.20 (0.92)	−0.02 (1.00)	0.02
**Episodic memory** ^b^	0.02 (0.99)	0.05 (0.99)	−0.19 (1.00)	0.04	0.09 (0.99)	−0.06 (0.99)	0.04	0.16 (0.92)	−0.01 (1.01)	0.07
**Semantic memory** ^b^	0.00 (0.99)	0.03 (0.98)	−0.18 (1.07)	0.09	0.09 (0.97)	−0.10 (1.00)	<0.01	0.32 (1.03)	−0.06 (0.97)	<0.01
**Average distance to facility (km)**	3.6 (2.8)	3.9 (2.8)	1.1 (0.3)	<0.01	4.8 (3.2)	2.1 (0.7)	<0.01	7.2 (5.0)	2.8 (1.1)	<0.01
**Total lead releases (lbs)** ^c^	4971.3 (6251.5)	5135.1 (6305.0)	3690.1 (5688.5)	0.03	5000.6 (6264.6)	4937.3 (6245.2)	0.89	4473.8 (6173.2)	5068.4 (6266.9)	0.33
**Air lead releases (lbs)**	27.1 (36.9)	27.8 (37.2)	21.6 (34.6)	0.12	26.9 (37.0)	27.1 (36.9)	0.96	23.8 (36.1)	27.7 (37.2)	0.28
**Water lead releases (lbs)**	0.8 (3.3)	0.8 (3.4)	0.4 (0.9)	<0.01	1.1 (4.3)	0.3 (0.9)	<0.01	2.5 (7.2)	0.4 (1.5)	<0.01
**Land lead releases (lbs)**	0.2 (4.6)	0.2 (4.9)	0.0 (0.0)	0.28	0.3 (6.3)	0.02 (0.4)	0.33	0.0 (0.0)	0.2 (5.1)	0.28
**PM_2.5_ (µg/m**3)	8.1 (2.1)	8.1 (2.1)	8.2 (2.4)	0.8	8.3 (2.0)	8.0 (2.3)	0.04	8.4 (2.3)	8.1 (2.1)	0.11
**Age at interview**	68.8 (8.8)	68.7 (8.8)	69.5 (9.3)	0.44	68.2 (8.7)	69.4 (8.9)	0.08	66.8 (8.2)	69.1 (8.9)	<0.01
**Sex**				0.99			0.57			0.39
Male	233 (31.4%)	207 (31.5%)	26 (31.0%)		129 (32.4%)	104 (30.3%)		42 (34.7%)	191 (30.8%)	
Female	508 (68.6%)	450 (68.5%)	58 (69.0%)		269 (67.6%)	239 (69.7%)		79 (65.3%)	429 (69.2%)	
**Education**				<0.01			0.03			0.01
≤ High school	135 (18.2%)	109 (16.6%)	26 (31%)		61 (15.3%)	74 (21.6%)		12 (9.9%)	123 (19.8%)	
> High school	606 (81.8%)	548 (83.4%)	58 (69%)		337 (84.7%)	269 (78.4%)		109 (90.1%)	497 (80.2%)	
**Marital status**				0.01			0.03			0.01
Married/ living as married	328 (44.3%)	302 (46.0%)	26 (31.0%)		191 (48.0%)	137 (39.9%)		66 (54.5%)	262 (42.3%)	
Not married	413 (55.7%)	355 (54.0%)	58 (69.0%)		207 (52.0%)	206 (60.1%)		55 (45.5%)	358 (57.7%)	
**Average census tract income**	103,014 (48,533)	105,122 (50,367)	86,530 (25,300)	<0.01	120,145 (57,805)	83,137 (21,911)	<0.01	147,727 (69,924)	94,288 (37,430)	<0.01
**Smoking status**				0.41			0.02			<0.01
Never	391 (52.8%)	352 (53.6%)	39 (46.4%)		227 (57.0%)	164 (47.8%)		79 (65.3%)	312 (50.3%)	
Past	307 (41.4%)	268 (40.8%)	39 (46.4%)		147 (36.9%)	160 (46.6%)		35 (28.9%)	272 (43.9%)	
Current	43 (5.8%)	37 (5.6%)	6 (7.1%)		24 (6.0%)	19 (5.5%)		7 (5.8%)	36 (5.8%)	
**Alcohol consumption**				0.27			0.01			<0.01
Never	270 (36.4%)	239 (36.4%)	31 (36.9%)		149 (37.4%)	121 (35.3%)		37 (30.6%)	233 (37.6%)	
Less than once a week	237 (32.0%)	210 (32.0%)	27 (32.1%)		110 (27.6%)	127 (37.0%)		30 (24.8%)	207 (33.4%)	
1–6 days per week	208 (28.1%)	182 (27.7%)	26 (31.0%)		120 (30.2%)	88 (25.7%)		46 (38.0%)	162 (26.1%)	
Every day	26 (3.5%)	26 (4.0%)	0.0 (0.0%)		19 (4.8%)	7 (2.0%)		8 (6.6%)	18 (2.9%)	

*Note*: Mean (SD) for continuous variables, *N* (%) categorical.

^a^ Fisher exact test, Satterthwaite *t* test.

^b^ Cognitive measures are *z* score standardized.

^c^ Total lead releases is a combination of air, land, water, and off‐site lead releases reported by the facility to the Toxics Release Inventory. Off‐site releases was not included in this analysis and therefore when combined, proportions of lead released through air, water, and land may not add to the total lead release number listed. Average census tract income rounded to the nearest whole number.

Abbreviations: km, kilometer; lbs, pounds; PM_2.5_, fine particulate matter with a diameter ≤ 2.5 micrometers; SD, standard deviation.

### 3.3 Residential distance to lead‐releasing facility association with cognitive domains

**FIGURE 3:**
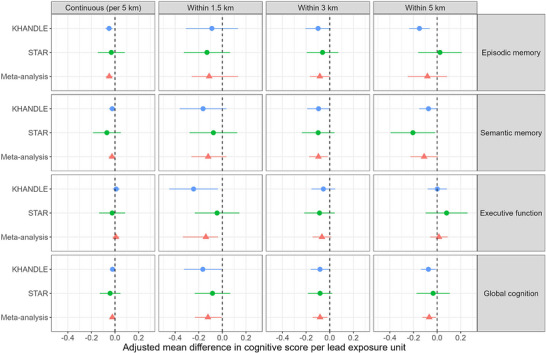
Forest plot of adjusted mean difference cognitive score and 95% confidence intervals by lead measure for KHANDLE (green) and STAR (red) analytic samples, and meta‐analyzed (blue) across cohorts. KHANDLE, Kaiser Healthy Aging and Diverse Life Experiences; STAR, xxx.

When examining bivariate associations with cognitive domain outcomes, participants living within any of the buffers (1.5 km, 3 km, 5 km) were more likely to have lower episodic memory, semantic memory, executive function, and global cognition scores (Tables S2 and S3 in supporting information). In KHANDLE fully adjusted models, for a residential address that was located closer to a lead‐releasing facility, episodic memory scores 2 years later were −0.05 standard deviation lower (95% confidence interval [CI]: −0.02, −0.08) per 5 km in continuous analyses (Figure 3 and Table S4 in supporting information). Living within 1.5 km of a lead‐releasing facility was associated with −0.24 standard deviation lower (95% CI: −0.45, −0.04) executive function scores, 2 years later. Living within 5 km of a lead‐releasing facility was associated with −0.15 standard deviation lower (95% CI: −0.24, −0.06) episodic memory scores, and −0.07 standard deviation lower (95% CI: −0.14, −0.01) global cognition scores, 2 years later. For comparison, a 1‐year increase in age was associated with −0.04 standard deviation lower (95% CI: −0.05, −0.04) episodic memory scores, and −0.04 standard deviation lower (95% CI: −0.05, −0.04) global cognition scores, 2 years later (Table S5 in supporting information). Across categorical exposure buffers, as buffer size increased, we observed slight attenuation of effect estimate. Semantic memory was not associated with continuous or categorical distance measures. Minimally adjusted models were generally stronger than the fully adjusted models (Figure S3 and Table S4 in supporting information).

In the STAR cohort, in fully adjusted models, living within 5 km of a lead‐releasing facility was associated with −0.20 standard deviation lower (95% CI: −0.39, −0.02) semantic memory scores, 2 years later (Figure 3 and Table S6 in supporting information). For comparison, a 1‐year increase in age was associated with −0.33 standard deviation lower (95% CI: −0.04, −0.03) semantic memory scores (Table S5). Episodic memory, executive function, and global cognition were not associated with either continuous or categorical distance exposures in fully adjusted models. Again, minimally adjusted models were generally stronger than fully adjusted models (Figure S3 and Table S6).

Combined meta‐analysis estimates showed similar magnitude and direction of effect with cohort‐specific main models (Table S7 in supporting information). In fully adjusted meta‐analysis models, for every 5 km closer a residence was located to a lead‐releasing facility, episodic memory scores 2 years later were −0.05 standard deviation lower (95% CI: −0.08, −0.02).

For comparison, in meta‐analyses, a 1‐year increase in age was associated with −0.04 standard deviation lower episodic memory (95% CI: −0.05, −0.04), −0.04 standard deviation lower semantic memory (95% CI: −0.04, −0.04), −0.05 standard deviation lower executive function (95% CI: −0.05, −0.04), and −0.04 standard deviation lower global cognition (95% CI: −0.05, −0.04).

### 3.4 Sensitivity analysis

Complete case descriptive statistics are available in Table S8 in supporting information. Overall, we observed associations with consistent magnitude with slight attenuation, compared to main models (Tables S9 and S10 in supporting information). In KHANDLE fully adjusted models, living within the 5 km exposure buffer was associated with −0.13 standard deviation lower (95% CI: −0.22, −0.05) episodic memory scores and −0.07 standard deviation lower (95% CI: −0.13, −0.002) global cognition scores, 2 years later. In STAR fully adjusted models, lower cognition was generally observed, but this only reached statistical significance with 5 km buffer exposure to a TRI facility and semantic memory (*β*:−0.19, 95% CI: −0.38, −0.004).

In age‐stratified models, the associations between residential distance to a lead‐releasing facility and lower cognitive domain scores remained consistent with main models (Tables S11 and S12 in supporting information). The largest magnitudes of association were observed for participants in the 70 to 74 and 75 to 79 age categories. In KHANDLE, for ages 70 to 74, living within 5 km of a lead‐releasing facility was associated with −0.23 standard deviation (95% CI: −0.39, −0.08) lower episodic memory scores, 2 years later. In STAR, for ages 75 to 79, living within 5 km of a lead‐releasing facility was associated with −0.54 standard deviation (95% CI: −1.05, −0.03) lower episodic memory scores, 2 years later.

For education‐stratified models, the magnitude and direction of associations were consistent with the main models (Tables S13 and S14 in supporting information). The largest magnitudes were observed for those reporting an education level of less than or equal to high school. In KHANDLE fully adjusted models among those with less than or equal to a high school education, living within the 5 km exposure buffer, lower cognitive scores were observed for episodic memory (*β*:−0.29, 95% CI: −0.51, −0.07), semantic memory (*β*:−0.36, 95% CI: −0.56, −0.15), and global cognition (*β*:−0.26, 95% CI: −0.43, −0.10). Among those with higher educational attainment, lead exposure was also associated with lower cognitive scores. In KHANDLE among those with a graduate degree, living within the 3 km buffer was associated with −0.27 standard deviation (95% CI: −0.50, −0.05) lower semantic memory scores, 2 years later, in fully adjusted models. In the STAR cohort, a similar trend was observed.

For street‐level sensitivity analyses, the exclusion of participants geocoded to city centroids did not significantly alter the primary association between distance to lead‐releasing facilities and cognition (Tables S15 and S16 in supporting information). In KHANDLE fully adjusted models, for the 5 km exposure buffer, beta estimates and confidence intervals maintained direction of effect with slight attenuation observed for episodic memory (*β*: −0.15, 95% CI: −0.24, −0.06), and remained the same for global cognition (*β*: −0.07, 95% CI: −0.13, −0.002) compared to main fully adjusted models. In STAR fully adjusted models, for the 5 km exposure buffer, a stronger association was observed for semantic memory (*β*:−0.24, 95% CI: −0.43, −0.05) compared to fully adjusted main models.

Descriptive statistics by residential buffer around a facility releasing ≥ 100 pounds of lead are available in Tables S17 and S18 in supporting information. Restricting analysis to facilities releasing ≥ 100 pounds of lead annually did not substantially impact the association between distance to lead‐releasing facilities and cognition compared to main models (Tables S19 and S20 in supporting information). For example, with the 5 km exposure buffer in KHANDLE, beta estimates and confidence intervals maintained direction of effect with slight attenuation observed for episodic memory (*β*: −0.11, 95% CI: −0.20, −0.20) compared to main models. Similarly, in STAR for the 5 km exposure buffer, we observed an attenuated association with a wider confidence interval for semantic memory (*β*:−0.10, 95% CI: −0.24, 0.05) compared to fully adjusted main models.

In sensitivity analysis stratified by mode of release (air, land, water) our findings were similar to main models (Table S21 in supporting information). For KHANDLE, with the 5 km air exposure buffer, beta estimates maintained direction of effect with slight attenuation for episodic memory (*β*: −0.13, 95% CI: −0.24, −0.03), with an increase in magnitude for semantic memory (*β*: −0.13, 95% CI: −0.23, −0.04) and global cognition (*β*: −0.13, 95% CI: −0.20, −0.04). In KHANDLE for the 5 km water exposure buffer, the magnitude of beta coefficient increased for episodic memory (*β*: −0.18, 95% CI: −0.33, −0.02), semantic memory (*β*: −0.17, 95% CI: −0.31, −0.03), executive function (*β*: −0.15, 95% CI: −0.30, −0.004), and global cognition (*β*: −0.16, 95% CI: −0.28, −0.05). In KHANDLE, there were 31 participants whose closest facility released ≥ 100 pounds of lead into the air, and in exploratory analyses, we observed associations with cognitive function (Table S21). For STAR the 5 km land exposure buffer beta estimates were larger in magnitude for semantic memory (*β*: −1.12, 95% CI: −2.10, −0.13). In STAR, continuous distance to a facility releasing > 0 pounds of lead by air had stronger magnitude of association and tighter confidence intervals, compared to main models for semantic memory scores (*β*: −0.23, CI: −0.43, −0.03; Table S21).

For mixed effects regression with a random intercept for census tract, results remained consistent with main models (Tables S22 and S23 in supporting information). No measurable residual correlation in outcomes among participants within the same census tract was observed. In KHANDLE for the 5 km exposure buffer, beta estimates were slightly attenuated for episodic memory (*β*: −0.12, 95% CI: −0.22, −0.03) and marginally larger for global cognition (*β*: −0.08, 95% CI: −0.14, −0.01) compared to main models. In STAR for the 5 km exposure buffer, the beta estimate remained the same with a slightly wider confidence interval for semantic memory (*β*: −0.20, 95% CI: −0.40, −0.003) compared to main models.

In extended analysis, adjusting for PM_2.5_ did not alter the primary association between distance to lead‐releasing facilities and cognition (Tables S24 and S25 in supporting information). The correlation between annual PM_2.5_ and continuous distance to a TRI facility releasing lead was −0.29 (*p* value < 0.0001) for KHANDLE, and −0.007 (*p* value 0.8) for STAR. In KHANDLE fully adjusted models, for the 5 km exposure buffer, beta estimates and confidence intervals maintained direction of effect with slight increase in magnitude observed for episodic memory (*β*: −0.16, 95% CI: −0.25, −0.07), and global cognition (*β*: −0.08, 95% CI: −0.15, −0.02) compared to main fully adjusted models. In STAR fully adjusted models, for the 5 km exposure buffer, a stronger association was observed for semantic memory (*β*: −0.21, 95% CI: −0.40, −0.02) compared to fully adjusted main models.

## 4 DISCUSSION

This study in two California longitudinal cohorts on aging found closer residential distance to lead‐releasing facilities was associated with worse cognitive scores 2 years later. We examined associations between residential distance to a lead‐releasing facility (continuously and buffers with radii of 1.5 km, 3 km, 5 km) and domain‐specific cognition 2 years later. Our most consistent finding, across cohorts and sensitivity analyses, was an association between residing within 5 km of a lead‐releasing facility and lower cognitive scores. In meta‐analyses, living within 5 km of a lead‐releasing facility was associated with −0.07 standard deviation (95% CI: −0.12, −0.0009) lower global cognition scores 2 years later. The direction of effect was consistent when we tested distance on a continuous scale and examined buffers of 1.5 km and 3 km. This study suggests residential lead exposure may be associated with lower cognitive performance and a potential modifiable risk factor for dementia.

Our findings align with work from the Cardiovascular Health Cognition Study (residences in Sacramento County, California; Washington County, Maryland; Forsyth County, North Carolina; Pittsburgh, Pennsylvania) demonstrating an association between industrial chemical releases and dementia risk in adults.^22^ That study used an area model of all TRI chemicals released to air or water weighted by their estimated toxicity called the Risk‐Screening Environmental Indicators (RSEI) model. Higher RSEI scores relate to higher concentrations of hazardous chemicals released.^40^ The Cardiovascular Health Cognition Study observed each RSEI score doubling was associated with 1.09 times higher odds of prevalent dementia (95% CI: 1.00, 1.19) and 1.16 times higher hazard of incident vascular dementia (95% CI: 1.01, 1.34).^22^ Past studies in children support our findings, reporting higher RSEI‐estimated toxic air emissions associated with worse cognitive performance.^41^, ^42^, ^43^ Other adverse health impacts have also been associated with nearby industrial facilities releasing toxic chemicals.^16^, ^41^, ^42^, ^43^, ^44^ These RSEI‐based prior works, like this study, use the same TRI data source. Our focus on lead was driven by our a priori hypothesis about lead toxicity on dementia‐related pathways, previous research, and the imperative to translate findings into actionable policy.^45^, ^46^

Most lead–dementia research has implemented biomarker‐based exposure assessments (i.e., blood lead) which reflect recent exposure rather than life‐long accumulated environmental burden.^16^, ^47^ Little is known about how geographically structured industrial exposures, like residential proximity to lead‐emitting facilities, affect cognitive aging in diverse populations. This limits the evaluation of long‐term environmental lead exposure in community‐based settings, especially in the context of environmental health disparities. Communities with higher proportions of historically racially marginalized populations have been well documented to experience disproportionate exposure to lead, particularly among children.^25^, ^26^ However, the evaluation of such inequities in adult populations remains understudied. This is particularly salient in California, where legacy industrial activity and demographic diversity create unique environmental and social exposure contexts. This study addressed these gaps by examining whether residential proximity to lead‐releasing facilities was associated with memory performance among older adults in California.

In the present analysis, closer residential distances to lead‐releasing facilities were associated with lower domain‐specific cognitive performance 2 years later. Findings suggest adulthood exposures may affect cognition and potentially contribute to dementia development. Results differed by exposure distance metric and cognitive domain, with episodic memory having the strongest associations across exposure distances. Potentially, cognitive domains may be differentially influenced by lead exposure. We observed lower cognitive scores among those with lower education, consistent with previous literature.^41^, ^48^, ^49^ However, we found no effect modification by education, indicating more education is not sufficient to mitigate the relationship between lead and cognition, as individuals with higher education also had a lower cognition association. Highlighting residential lead exposure in adulthood is both an environmental health concern and environmental justice concern. To our knowledge, this study is the first to leverage TRI facility data to assess associations with adult domain‐specific cognition.

Lead is a well‐established neurotoxicant that can cross the blood–brain barrier and accumulate in brain tissue.^50^ Once in the central nervous system, it alters neurophysiological processes through multiple pathways, including disrupting neurotransmission, promoting oxidative stress, compromising membrane integrity, and mimicking calcium ions (Ca^2^^+^) to impair intracellular signaling and synaptic activity.^50^, ^51^, ^52^, ^53^ These biological disruptions, combined with lead's role in inducing neuroinflammation, create a pathological environment that may accelerate cognitive decline. The impact of lead on cognitive function may occur through direct neurotoxic mechanisms and indirect pathways.^46^, ^47^, ^50^ For example, lead‐induced hypertension and cardiovascular dysfunction have been linked to cognitive impairment and dementia.^47^, ^51^ Biological evidence strongly supports a mechanistic link between lead exposure and neurodegeneration. However, epidemiologic evidence in older adult populations with environmentally relevant exposures remains limited, particularly using geospatial methods to characterize long‐term environmental burden.^22^

### 4.1 Limitations and future directions

Our analysis has several limitations. The STAR cohort sample size may limit the ability to detect associations between exposure and outcome measures. Also, distance measures were used as a proxy for exposure, which may introduce exposure misclassification, as distance measures may not reflect actual exposure levels and assume spatial homogeneity of exposure. However, distance‐based models are considered appropriate when the disease etiology is unclear and provide beneficial exploratory analysis to guide more sophisticated future analyses.^10^, ^44^, ^54^, ^55^ In addition, elevated blood lead levels have been associated with proximity to airports, roadways, and TRI sites, providing evidence for the validity of our distance‐based exposure metric.^11^, ^13^, ^15^, ^16^ TRI facilities releasing lead may release other neurotoxicants, so proximity to these sites may also represent proximity to other toxic releases. Although we focused on TRI facilities that released lead from 2015 to 2018, living near these facilities could have exposed people to decades of chronic toxic releases. We assessed prior lead exposure at one time point, which does not reflect the true lifetime lead exposure for participants.

The exposome encompasses the totality of factors a person encounters throughout their lifetime such as behavioral, biological, and environmental exposures, of which lead is an important chemical component.^56^ We encourage forthcoming research to build upon this study's findings by exploring additional exposomic components. Future analyses may include neurotoxic chemical mixtures such as lead, mercury, and cadmium, or subgroup analysis by demographic and comorbid factors. Moreover, studies incorporating information on prevailing wind direction, which influences where and how pollutants travel from their sources, would prove a more accurate picture of exposure and health consequences.^57^ Further, longitudinal analysis with repeated cognitive measures and cumulative exposures would greatly benefit the growing body of exposome research.

### 4.2 Strengths

A significant strength of this study is the diverse population, allowing us to study exposure effects in largely underrepresented populations who were enrolled in health‐care plans and had equal access to health care. We build on the important body of air pollution and dementia research by examining industrial releases as an upstream source of pollution that may provide intervention opportunities. In addition, our 2‐year lagged study design between exposure and cognitive measure provides information on how lead exposure impacts domain‐specific cognition over relatively short time periods. Extensive sensitivity analysis demonstrated strong consistency and stability of our results. Findings persisted after restricting analysis to a higher threshold of lead release. Results were robust across release media types, with the greatest number of significant associations observed for releases in air and water. When subjected to a threshold of air release, results remained consistent. Analyses using mixed effects models with a random intercept for census tract showed the observed differences in cognitive outcomes are likely explained by individual factors, rather than shared census tract characteristics. The consistency of effect estimates across main models and extended models adjusting for annual PM_2.5_ suggests confounding by annual PM_2.5_ is unlikely to be a major source of bias in this study. Finally, this analysis helps fill the knowledge gap concerning how cumulative adult lead exposure influences cognitive decline and dementia risk.  Our findings, together with the literature, indicate adult and childhood lead exposures may be important factors to consider when assessing lifetime dementia risk.

### 4.3 Summary and implications

In this analysis, closer residential proximity to lead‐releasing facilities was associated with lower episodic memory, semantic memory, executive function, and global cognition, 2 years before cognitive testing. Based on our findings, living within 5 km of a lead‐releasing facility was equivalent to the impact of 0.5 to 3 years of aging on episodic memory and 1.8 to 6.6 years of aging on semantic memory. Because aging is the primary predictor for dementia and cognitive impairment among older adults, the finding that lead exposure exhibits comparable effect sizes to aging underscores the public health concern. In addition, while these differences in absolute cognitive scores may seem small, the high prevalence of exposure (45% living within 5 km in KHANDLE, 83% in STAR) suggests the population attributable fraction could be substantial and interventions to reduce industrial releases near residences could yield a considerable public health benefit. Society‐wide interventions to reduce lead exposure include tightening regulatory controls on industrial production and systematic abatement of environmental lead contamination. Individual‐level changes to reduce lead exposure include adequate iron and calcium intake and if living in housing built before 1978, regularly cleaning windowsills and floors, covering chipping paint, and avoiding projects creating lead paint dust.^58^ Policies and individual practices to reduce lead exposure may be effective for preventing cognitive impairment among older adults.

## CONFLICT OF INTEREST STATEMENT

The authors declare no conflicts of interest. Author disclosures are available in the Supporting Information.

## CONSENT STATEMENT

All study participants provided written informed consent.

## Supporting information




Supporting Information

Supporting Information

## Data Availability

All lead exposure data used for this analysis are publicly available and can be downloaded from the EPA TRI Toxics Tracker webpage at https://edap.epa.gov/public/extensions/TRIToxicsTracker/TRIToxicsTracker.html. For access to participant data used in this analysis, please contact the senior author, Kathryn Conlon, kcconlon@health.ucdavis.edu.
