# Supplementary material for: Association between proximity to a lead‐releasing facility and cognition in diverse cohorts
Source: Alzheimers Dement. 2026 May 1;22(5):e71446. doi: 10.1002/alz.71446 (PMC13133548; doi:10.1002/alz.71446)
Supplement: Supplementary file 2 — Supporting Information [file ALZ-22-e71446-s002.docx]

**Title:** Association between proximity to a lead releasing facility and cognition in diverse cohorts

Supplemental tables: 25; Supplemental figures: 3.

**
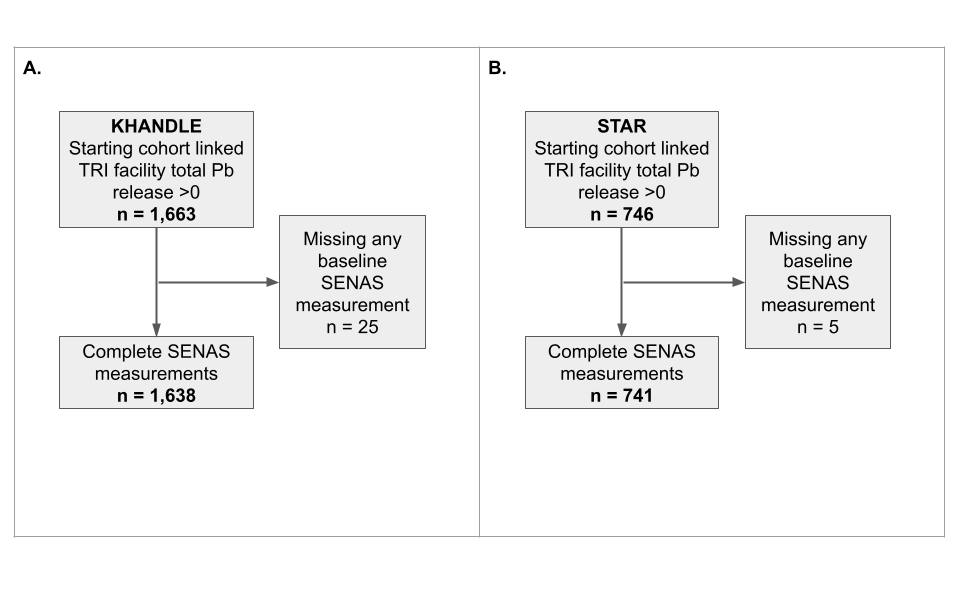
**

**Supplemental Figure 1**. Flowchart for study inclusion. Panel A represents selection for Kaiser Healthy Aging and Diverse Life Experiences Study (KHANDLE). Panel B represents selection for Study of Healthy Aging in African Americans (STAR). SENAS: Spanish and English neuropsychological assessment scales; TRI: Toxics release inventory; Pb: lead.

**Supplementary Table 1.** Descriptive statistics for the included and excluded participants in the Kaiser Healthy Aging and Diverse Life Experiences Study (KHANDLE) and Study of Healthy Aging in African Americans (STAR) cohorts.

|  | **Cohort** | | | | | | | |
| --- | --- | --- | --- | --- | --- | --- | --- | --- |
|  | **KHANDLE** | | | | **STAR** | | | |
| **Characteristic** | **Overall**  N = 1,663 | **Excluded**  N = 25 | **Included**  N = 1,638 | ***p* value*^1^*** | **Overall,**  N = 746 | **Excluded,**  N = 5 | **Included,**  N = 741 | ***p* value*^1^*** |
| **Global cognition^2^** | 0.01 (0.81) | 0.79 (0.00) | 0.01 (0.81) | 0.07 | 0.01 (0.81) | - | 0.01 (0.81) | 0.02 |
| (missing) | 1 | 1 | - |  |  |  |  |  |
| **Executive function^2^** | 0.01 (1.00) | -0.28 (0.99) | 0.01 (1.00) | <0.01 | 0.01 (0.99) | -1.15 (1.22) | 0.01 (0.99) | <0.01 |
| (missing) | 10 | 10 | - |  |  |  |  |  |
| **Episodic memory^2^** | 0.01 (1.00) | -0.05 (0.94) | 0.01 (1.00) | 0.60 | 0.02 (0.99) | -0.46 (0.65) | 0.02 (0.99) | 0.08 |
| (missing) | 4 | 4 | - |  | 2 | 2 | - |  |
| **Semantic memory^2^** | 0.01 (1.00) | -0.40 (1.39) | 0.02 (0.99) | 0.06 | 0.00 (0.99) | -0.09 (0.42) | 0.00 (0.99) | 0.03 |
| (missing) | 7 | 7 | - |  | 3 | 3 | - |  |
| **Average distance to facility (km)** | 8.2 (6.9) | 7.4 (6.8) | 8.2 (6.9) | 0.36 | 3.6 (2.8) | 3.0 (1.7) | 3.6 (2.8) | 0.44 |
| **Lead releasing facility within 1.5km** |  |  |  | 0.19 |  |  |  | 0.34 |
| No | 1602 (96.3%) | 24 (96.0%) | 1578 (96.3%) |  | 661 (88.6%) | 4 (80.0%) | 657 (88.7%) |  |
| Yes | 61 (3.7%) | 1 (4.0%) | 60 (3.7%) |  | 85 (11.4%) | 1 (20.0%) | 84 (11.3%) |  |
| **Lead releasing facility within 3km** |  |  |  | 0.46 |  |  |  | 0.16 |
| No | 1331 (80.0%) | 19 (76.0%) | 1312 (80.1%) |  | 401 (53.8%) | 3 (60.0%) | 398 (53.7%) |  |
| Yes | 332 (20.0%) | 6 (24.0%) | 326 (19.9%) |  | 345 (46.2%) | 2 (40.0%) | 343 (46.3%) |  |
| **Lead releasing facility within 5km** |  |  |  | 0.23 |  |  |  | 0.16 |
| No | 907 (54.5%) | 11 (44.0%) | 896 (54.7%) |  | 122 (16.4%) | 1 (20.0%) | 121 (16.3%) |  |
| Yes | 756 (45.5%) | 14 (56.0%) | 742 (45.3%) |  | 624 (83.6%) | 4 (80.0%) | 620 (83.7%) |  |
| **Total lead releases (lbs)^3^** | 2,307.5 (5,331.9) | 1,908.6 (4,516.4) | 2,313.6 (5,344.3) | 0.90 | 4,956.1 (6,245.5) | 2,712.0 (5,351.4) | 4,971.3 (6,251.5) | 0.22 |
| **Air lead releases (lbs)** | 11.3 (30.1) | 20.5 (47.6) | 11.1 (29.8) | 0.30 | 26.9 (36.9) | 17.1 (38.2) | 27.1 (36.9) | 0.29 |
| **Water lead releases (lbs)** | 1.5 (7.2) | 0.4 (2.2) | 1.6 (7.2) | 0.56 | 0.8 (3.3) | 0.0 | 0.8 (3.3) | <0.01 |
| **Land lead releases (lbs)** | 10.2 (230.7) | 0.0 | 10.4 (232.5) | 0.07 | 0.2 (4.6) | 0.0 | 0.2 (4.6) | 0.28 |
| **Age at interview** | 76.1 (7.2) | 80.3 (8.9) | 76.1 (7.1) | 0.01 | 68.8 (8.8) | 69.5 (5.7) | 68.8 (8.8) | 0.50 |
| **Sex** |  |  |  | 0.90 |  |  |  | 0.19 |
| Male | 684 (41.1%) | 10 (40.0%) | 674 (41.1%) |  | 235 (31.5%) | 2 (40.0%) | 233 (31.4%) |  |
| Female | 979 (58.9%) | 15 (60.0%) | 964 (58.9%) |  | 511 (68.5%) | 3 (60.0%) | 508 (68.6%) |  |
| **Race/ethnicity** |  |  |  | <0.01 |  |  |  | <0.01 |
| Asian | 412 (24.8%) | 2 (8.0%) | 410 (25.0%) |  | - | - | - |  |
| Black | 431 (25.9%) | 9 (36.0%) | 422 (25.8%) |  | 736 (98.7%) | 5 (100.0%) | 731 (98.7%) |  |
| LatinX | 327 (19.7%) | 5 (20.0%) | 322 (19.7%) |  | 6 (0.8%) | - | 6 (0.8%) |  |
| Native American | 3 (0.2%) | 3 (12.0%) | - |  | 4 (0.5%) | - | 4 (0.5%) |  |
| White | 490 (29.5%) | 6 (24.0%) | 484 (29.5%) |  | - | - | - |  |
| **Education** |  |  |  | <0.01 |  |  |  | <0.01 |
| ≤ High School | 281 (16.9%) | 7 (28.0%) | 274 (16.7%) |  | 136 (18.2%) | 1 (20.0%) | 135 (18.2%) |  |
| > High School | 1382 (83.1%) | 18 (72.0%) | 1364 (83.3%) |  | 610 (81.8%) | 4 (80.0%) | 606 (81.8%) |  |
| **Marital Status** |  |  |  | <0.01 |  |  |  | <0.01 |
| Married/ living as married | 944 (56.8%) | 11 (44.0%) | 933 (57.0%) |  | 329 (44.1%) | 1 (20.0%) | 328 (44.3%) |  |
| Not Married | 714 (42.9%) | 9 (36.0%) | 705 (43.0%) |  | 416 (55.8%) | 3 (60.0%) | 413 (55.7%) |  |
| (missing) | 5 | 5 | - |  | 1 | 1 | - |  |
| **Average census tract income** | 1,213,78 (52,401) | 123,999 (59,546) | 121,338 (52,304) | 0.68 | 103,178 (48,673) | 127,476 (688,045) | 103,014 (48,533) | 0.81 |
| **Smoking status** |  |  |  | <0.01 |  |  |  | <0.01 |
| Never | 920 (55.3%) | 8 (32.0%) | 912 (55.7%) |  | 396 (53.1%) | 5 (100%) | 391 (52.8%) |  |
| Former | 691 (41.6%) | 14 (56.0%) | 677 (41.3%) |  | 307 (41.2%) | - | 307 (41.4%) |  |
| Current | 52 (3.1%) | 3 (12.0%) | 49 (3.0%) |  | 43 (5.8%) | - | 43 (5.8%) |  |
| **Alcohol consumption** |  |  |  | <0.01 |  |  |  | <0.01 |
| Never | 492 (29.6%) | 11 (44.0%) | 481 (29.4%) |  | 274 (36.7%) | 4 (80%) | 270 (36.4%) |  |
| Less than once a week | 580 (34.9%) | 8 (32.0%) | 572 (34.9%) |  | 237 (31.8%) | - | 237 (32.0%) |  |
| 1-6 days per week | 430 (25.9%) | 5 (20.0%) | 425 (25.9%) |  | 208 (27.9%) | - | 208 (28.1%) |  |
| Every day | 161 (9.7%) | 1 (4.0%) | 160 (9.8%) |  | 27 (3.6%) | 1 (20%) | 26 (3.5%) |  |

Mean (SD) for continuous variables, N(%) categorical. ^1^Fishers exact test, Satterthwaite t-test. ^2^Cognitive measures are z-score standardized. ^3^Total lead releases is a combination of air, land, water, and off-site lead releases reported by the facility to the Toxics Release Inventory. Off-site releases was not included in this analysis and therefore when combined, proportions of lead released through air, water, and land may not add to the total lead release number listed. Average census tract income rounded to the nearest whole number. lbs: pounds, km: kilometers


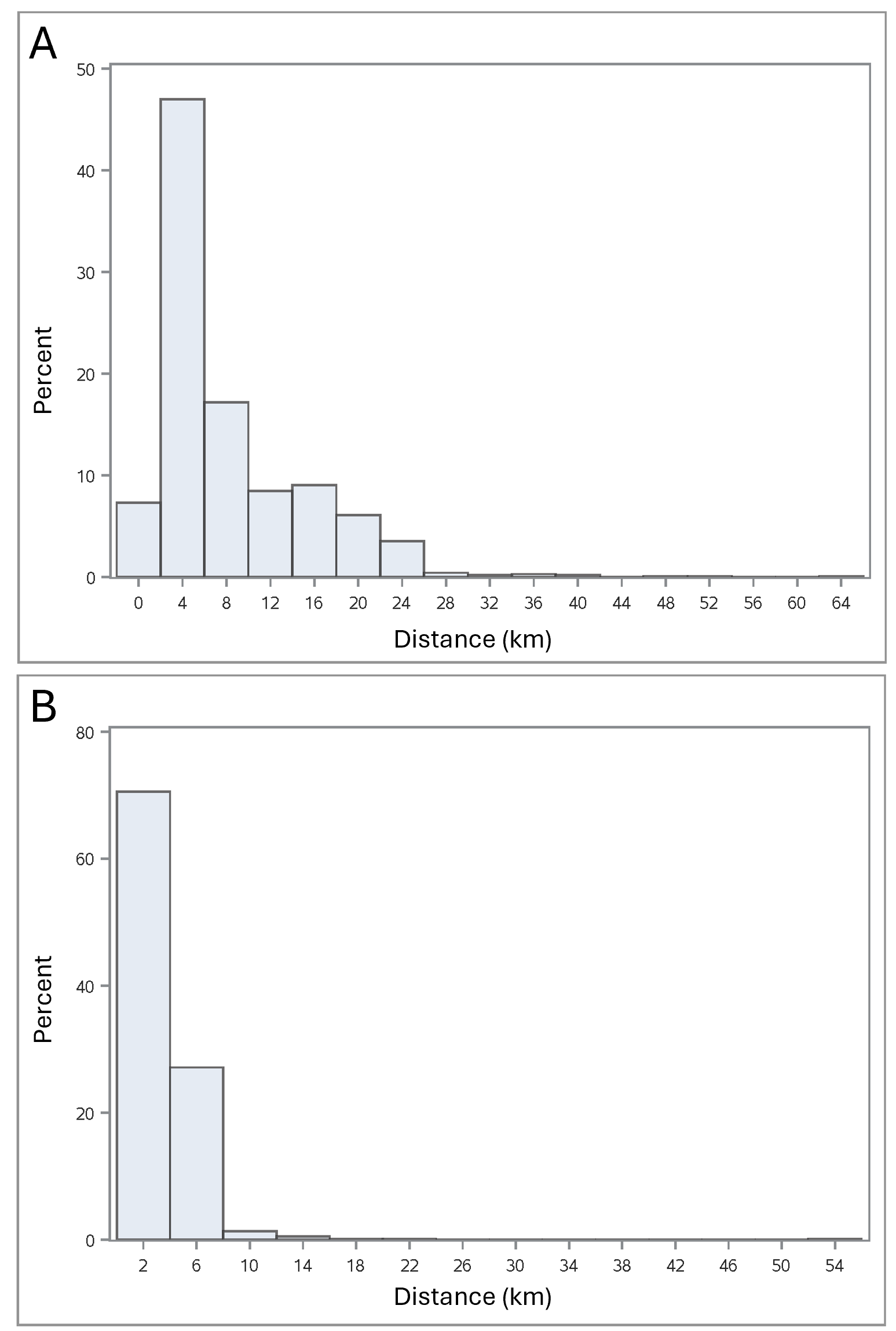


**Supplemental Figure 2.** Distributions of residential distance to lead releasing facility in kilometers (km) in analytic sample by cohort. Panel A represents Kaiser Healthy Aging and Diverse Life Experiences Study (KHANDLE). Panel B represents Study of Healthy Aging in African Americans (STAR). km: kilometers.

**Supplemental Table 2.** Kaiser Healthy Aging and Diverse Life Experiences Study (KHANDLE) analytic descriptive statistics by baseline cognitive domain test results.

|  |  | **Cognitive domain tested** | | | | | | | | | | | |
| --- | --- | --- | --- | --- | --- | --- | --- | --- | --- | --- | --- | --- | --- |
| **Characteristic** | **Overall**  N = 1,638 | **Global cognition** | | | **Executive functioning** | | | **Episodic memory** | | | **Semantic memory** | | |
|  |  | **Low**  N = 783 | **High**  N = 855 | ***p* value*^1^*** | **Low**  N = 820 | **High**  N = 818 | ***p* value*^1^*** | **Low**  N =808 | **High**  N = 830 | ***p* value*^1^*** | **Low**  N = 764 | **High**  N = 874 | ***p* value*^1^*** |
| **Average distance to facility (km)** | 8.2 (6.9) | 7.8 (7.1) | 8.6 (6.8) | 0.03 | 8 (6.8) | 8.4 (7) | 0.29 | 7.9 (7.1) | 8.5 (6.7) | 0.05 | 7.6 (6.5) | 8.8 (7.2) | <0.01 |
| **Lead facility within 1.5 km** |  |  |  | 0.18 |  |  | 0.08 |  |  | 0.89 |  |  | 0.06 |
| No | 1578 (96.3%) | 749 (95.7%) | 829 (97.0%) |  | 783 (95.5%) | 795 (97.2%) |  | 779 (96.4%) | 799 (96.3%) |  | 729 (95.4%) | 849 (97.1%) |  |
| Yes | 60 (3.7%) | 34 (4.3%) | 26 (3.0%) |  | 37 (4.5%) | 23 (2.8%) |  | 29 (3.6%) | 31 (3.7%) |  | 35 (4.6%) | 25 (2.9%) |  |
| **Lead facility within 3 km** |  |  |  | <0.01 |  |  | <0.01 |  |  | 0.10 |  |  | <0.01 |
| No | 1312 (80.1%) | 605 (77.3%) | 707 (82.7%) |  | 635 (77.4%) | 677 (82.8%) |  | 634 (78.5%) | 678 (81.7%) |  | 579 (75.8%) | 733 (83.9%) |  |
| Yes | 326 (19.9%) | 178 (22.7%) | 148 (17.3%) |  | 185 (22.6%) | 141 (17.2%) |  | 174 (21.5%) | 152 (18.3%) |  | 185 (24.2%) | 141 (16.1%) |  |
| **Lead facility within 5 km** |  |  |  | <0.01 |  |  | 0.04 |  |  | 0.01 |  |  | <0.01 |
| No | 896 (54.7%) | 393 (50.2%) | 503 (58.8%) |  | 428 (52.2%) | 468 (57.2%) |  | 417 (51.6%) | 479 (57.7%) |  | 376 (49.2%) | 520 (59.5%) |  |
| Yes | 742 (45.3%) | 390 (49.8%) | 352 (41.2%) |  | 392 (47.8%) | 350 (42.8%) |  | 391 (48.4%) | 351 (42.3%) |  | 388 (50.8%) | 354 (40.5%) |  |
| **Total lead releases (lbs)^3^** | 2,313.6 (5,344.3) | 2,811.7 (6,111.4) | 1,857.5 (4,484.8) | <0.01 | 2,640.8 (5,988.9) | 1,985.6 (4,588.9) | 0.01 | 2,644.4 (5,572.9) | 1,991.6 (5,094.7) | 0.01 | 2,794.9 (6,111.8) | 1,892.8 (4,531.0) | <0.01 |
| **Air lead releases (lbs)** | 11.1 (29.8) | 13.3 (31.9) | 9.1 (27.5) | <0.01 | 12.2 (30.4) | 10.1 (29.1) | 0.14 | 12.2 (30.5) | 10.1 (29.1) | 0.15 | 13.9 (32.2) | 8.6 (27.2) | <0.01 |
| **Water lead releases (lbs)** | 1.6 (7.2) | 1.6 (7.2) | 1.5 (7.3) | 0.72 | 1.7 (7.8) | 1.4 (6.7) | 0.30 | 1.5 (6.9) | 1.6 (7.6) | 0.84 | 1.8 (7.8) | 1.3 (6.7) | 0.15 |
| **Land Lead releases (lbs)** | 10.4 (232.5) | 4.9 (104.9) | 15.3 (305.7) | 0.35 | 1.5 (17.3) | 19.3 (328.4) | 0.12 | 19.3 (330.4) | 1.6 (17.6) | 0.12 | 4.5 (105.7) | 15.5 (302.5) | 0.31 |
| **PM_2.5_ (μg/m^3^)** | 7.5 (1.5) | 7.4 (1.4) | 7.5 (1.5) | 0.48 | 7.4 (1.4) | 7.5 (1.5) | 0.86 | 7.4 (1.5) | 7.5 (1.4) | 0.42 | 7.4 (1.5) | 7.5 (1.5) | 0.44 |
| **Age at interview** | 76.1 (7.1) | 78.6 (7.6) | 73.7 (5.8) | <0.01 | 78.2 (7.6) | 73.9 (5.9) | <0.01 | 78.3 (7.6) | 73.9 (5.9) | <0.01 | 78.0 (7.6) | 74.4 (6.2) | <0.01 |
| **Sex** |  |  |  | <0.01 |  |  | <0.01 |  |  | <0.01 |  |  | <0.01 |
| Male | 674 (41.1%) | 349 (44.6%) | 325 (38.0%) |  | 383 (46.7%) | 291 (35.6%) |  | 419 (51.9%) | 255 (30.7%) |  | 251 (32.9%) | 423 (48.4%) |  |
| Female | 964 (58.9%) | 434 (55.4%) | 530 (62.0%) |  | 437 (53.3%) | 527 (64.4%) |  | 389 (48.1%) | 575 (69.3%) |  | 513 (67.1%) | 451 (51.6%) |  |
| **Race/ethnicity** |  |  |  | <0.01 |  |  | <0.01 |  |  | <0.01 |  |  | <0.01 |
| Asian | 410 (25.0%) | 211 (26.9%) | 199 (23.3%) |  | 230 (28.0%) | 180 (22.0%) |  | 169 (20.9%) | 241 (29.0%) |  | 229 (30.0%) | 181 (20.7%) |  |
| Black | 422 (25.8%) | 265 (33.8%) | 157 (18.4%) |  | 254 (31.0%) | 168 (20.5%) |  | 234 (29.0%) | 188 (22.7%) |  | 299 (39.1%) | 123 (14.1%) |  |
| LatinX | 322 (19.7%) | 172 (22.0%) | 150 (17.5%) |  | 193 (23.5%) | 129 (15.8%) |  | 185 (22.9%) | 137 (16.5%) |  | 137 (17.9%) | 185 (21.2%) |  |
| White | 484 (29.5%) | 135 (17.2%) | 349 (40.8%) |  | 143 (17.4%) | 341 (41.7%) |  | 220 (27.2%) | 264 (31.8%) |  | 99 (13.0%) | 385 (44.1%) |  |
| **Education** |  |  |  | <0.01 |  |  | <0.01 |  |  | <0.01 |  |  | <0.01 |
| ≤ High School | 274 (16.7%) | 206 (26.3%) | 68 (8.0%) |  | 206 (25.1%) | 68 (8.3%) |  | 195 (24.1%) | 79 (9.5%) |  | 190 (24.9%) | 84 (9.6%) |  |
| > High School | 1364 (83.3%) | 577 (73.7%) | 787 (92.0%) |  | 614 (74.9%) | 750 (91.7%) |  | 613 (75.9%) | 751 (90.5%) |  | 574 (75.1%) | 790 (90.4%) |  |
| **Marital Status** |  |  |  | <0.01 |  |  | <0.01 |  |  | <0.01 |  |  | <0.01 |
| Married/ living as married | 933 (57.0%) | 399 (51.0%) | 534 (62.5%) |  | 434 (52.9%) | 499 (61.0%) |  | 442 (54.7%) | 491 (59.2%) |  | 349 (45.7%) | 584 (66.8%) |  |
| Not married | 705 (43.0%) | 384 (49.0%) | 321 (37.5%) |  | 386 (47.1%) | 319 (39.0%) |  | 366 (45.3%) | 339 (40.8%) |  | 415 (54.3%) | 290 (33.2%) |  |
| **Average census tract income** | 121,338 (52,304) | 112,688 (4,9341) | 129,259 (53,696) | <0.01 | 114,432 (49,795) | 128,260 (53,852) | <0.01 | 117,017 (53,309) | 125,544 (50,987) | <0.01 | 112,006 (47,631) | 129,495 (54,810) | <0.01 |
| **Smoking status** |  |  |  | 0.74 |  |  | 0.65 |  |  | 0.01 |  |  | 0.74 |
| Never | 912 (55.7%) | 433 (55.3%) | 479 (56.0%) |  | 466 (56.8%) | 446 (54.5%) |  | 424 (52.5%) | 488 (58.8%) |  | 444 (58.1%) | 468 (53.5%) |  |
| Past | 677 (41.3%) | 324 (41.4%) | 353 (41.3%) |  | 330 (40.2%) | 347 (42.4%) |  | 354 (43.8%) | 323 (38.9%) |  | 298 (39.0%) | 379 (43.4%) |  |
| Current | 49 (3.0%) | 26 (3.3%) | 23 (2.7%) |  | 24 (2.9%) | 25 (3.1%) |  | 30 (3.7%) | 19 (2.3%) |  | 22 (2.9%) | 27 (3.1%) |  |
| **Alcohol consumption** |  |  |  | <0.01 |  |  | <0.01 |  |  | <0.01 |  |  | <0.01 |
| Never | 481 (29.4%) | 294 (37.5%) | 187 (21.9%) |  | 291 (35.5%) | 190 (23.2%) |  | 275 (34.0%) | 206 (24.8%) |  | 295 (38.6%) | 186 (21.3%) |  |
| Less than once a week | 572 (34.9%) | 266 (34.0%) | 306 (35.8%) |  | 296 (36.1%) | 276 (33.7%) |  | 247 (30.6%) | 325 (39.2%) |  | 278 (36.4%) | 294 (33.6%) |  |
| 1 - 6 days per week | 425 (25.9%) | 150 (19.2%) | 275 (32.2%) |  | 164 (20.0%) | 261 (31.9%) |  | 196 (24.3%) | 229 (27.6%) |  | 138 (18.1%) | 287 (32.8%) |  |
| Every day | 160 (9.8%) | 73 (9.3%) | 87 (10.2%) |  | 69 (8.4%) | 91 (11.1%) |  | 90 (11.1%) | 70 (8.4%) |  | 53 (6.9%) | 107 (12.2%) |  |

Mean (SD) for continuous variables, N(%) categorical. ^1^Fishers exact test, Satterthwaite t-test. ^2^Cognitive measures are z-score standardized. ^3^Total lead releases is a combination of air, land, water, and off-site lead releases reported by the facility to the Toxics Release Inventory. Off-site releases were not included in this analysis and therefore when combined, proportions of lead released through air, water, and land may not add to the total lead release number listed. Average census tract income rounded to the nearest whole number. Each cognitive outcome, dichotomized into “high” and “low” based on a cut-point of zero. lbs: pounds, km: kilometers, PM_2.5_: atmospheric particulate matter with a diameter < 2.5 micrometers.

**Supplemental Table 3.** Study of Healthy Aging in African Americans (STAR) analytic sample descriptive statistics by baseline cognitive domain test results.

|  |  | **Cognitive measure** | | | | | | | | | | |  |
| --- | --- | --- | --- | --- | --- | --- | --- | --- | --- | --- | --- | --- | --- |
| **Characteristic** | **Overall**  N = 741 | **Global cognition** | |  | **Executive functioning** | |  | **Episodic memory** | |  | **Semantic memory** | |  |
|  |  | **Low**  N = 341 | **High**  N = 400 | ***p* value*^1^*** | **Low**  N = 354 | **High**  N = 387 | ***p* value*^1^*** | **Low**  N = 358 | **High**  N = 383 | ***p* value*^1^*** | **Low**  N = 350 | **High**  N = 391 | ***p* value*^1^*** |
| **Average distance to facility (km)** | 3.6 (2.8) | 3.3 (1.9) | 3.8 (3.3) | 0.02 | 3.3 (1.9) | 3.8 (3.4) | <0.01 | 3.4 (1.9) | 3.7 (3.4) | 0.05 | 3.5 (3.3) | 3.7 (2.2) | 0.34 |
| **Lead facility within 1.5 km** |  |  |  | 0.03 |  |  | 0.04 |  |  | 0.03 |  |  | 0.06 |
| No | 657 (88.7%) | 293 (85.9%) | 364 (91.0%) |  | 305 (86.2%) | 352 (91.0%) |  | 308 (86.0%) | 349 (91.1%) |  | 302 (86.3%) | 355 (90.8%) |  |
| Yes | 84 (11.3%) | 48 (14.1%) | 36 (9.0%) |  | 49 (13.8%) | 35 (9.0%) |  | 50 (14.0%) | 34 (8.9%) |  | 48 (13.7%) | 36 (9.2%) |  |
| **Lead facility within 3 km** |  |  |  | 0.05 |  |  | <0.01 |  |  | 0.05 |  |  | 0.20 |
| No | 398 (53.7%) | 170 (49.9%) | 228 (57.0%) |  | 170 (48.0%) | 228 (58.9%) |  | 179 (50.0%) | 219 (57.2%) |  | 179 (51.1%) | 219 (56.0%) |  |
| Yes | 343 (46.3%) | 171 (50.1%) | 172 (43.0%) |  | 184 (52.0%) | 159 (41.1%) |  | 179 (50.0%) | 164 (42.8%) |  | 171 (48.9%) | 172 (44.0%) |  |
| **Lead facility within 5 km** |  |  |  | 0.03 |  |  | 0.01 |  |  | 0.23 |  |  | 0.04 |
| No | 121 (16.3%) | 45 (13.2%) | 76 (19.0%) |  | 45 (12.7%) | 76 (19.6%) |  | 52 (14.5%) | 69 (18.0%) |  | 47 (13.4%) | 74 (18.9%) |  |
| Yes | 620 (83.7%) | 296 (86.8%) | 324 (81.0%) |  | 309 (87.3%) | 311 (80.4%) |  | 306 (85.5%) | 314 (82.0%) |  | 303 (86.6%) | 317 (81.1%) |  |
| **Total lead releases (lbs)^3^** | 4,971.3 (6,251.5) | 5,116.9 (6,306.9) | 4,847.1 (6,208.9) | 0.55 | 5,230.8 (6,300.3) | 4,733.9 (6,205.1) | 0.28 | 4,836.9 (6,245.1) | 5,096.9 (6,262.9) | 0.57 | 5,029.4 (6,267.2) | 4,919.2 (6,244.9) | 0.81 |
| **Air lead releases (lbs)** | 27.1 (36.9) | 28.8 (38.0) | 25.6 (36.0) | 0.24 | 29.3 (38.3) | 25.0 (35.7) | 0.11 | 26.9 (37.4) | 27.2 (36.6) | 0.92 | 27.9 (37.6) | 26.3 (36.5) | 0.54 |
| **Water lead releases (lbs)** | 0.8 (3.3) | 0.7 (2.7) | 0.8 (3.7) | 0.52 | 0.7 (2.4) | 0.9 (3.9) | 0.39 | 0.8 (3.3) | 0.8 (3.2) | 0.96 | 0.7 (2.7) | 0.8 (3.7) | 0.53 |
| **Land lead releases (lbs)** | 0.2 (4.6) | 0.4 (6.8) | 0.03 (0.4) | 0.35 | 0.0 (0.0) | 0.4 (6.4) | 0.28 | 0.0 (0.0) | 0.4 (6.5) | 0.28 | 0.4 (6.7) | 0.02 (0.4) | 0.33 |
| **PM_2.5_ (μg/m^3^)** | 8.1 (2.1) | 8.2 (2.2) | 8.1 (2.1) | 0.48 | 8.1 (2.3) | 8.1 (2.0) | 0.91 | 8.2 (2.2) | 8.1 (2.1) | 0.81 | 8.2 (2.2) | 8.1 (2.1) | 0.28 |
| **Age at interview** | 68.8 (8.8) | 72.6 (9.4) | 65.5 (6.8) | <0.01 | 72.4 (9.1) | 65.4 (7.1) | <0.01 | 71.9 (9.3) | 65.8 (7.1) | <0.01 | 71.3 (9.6) | 66.5 (7.4) | <0.01 |
| **Sex** |  |  |  | <0.01 |  |  | <0.01 |  |  | <0.01 |  |  | 0.02 |
| Male | 233 (31.4%) | 126 (37.0%) | 107 (26.8%) |  | 134 (37.9%) | 99 (25.6%) |  | 153 (42.7%) | 80 (20.9%) |  | 95 (27.1%) | 138 (35.3%) |  |
| Female | 508 (68.6%) | 215 (63.0%) | 293 (73.3%) |  | 220 (62.1%) | 288 (74.4%) |  | 205 (57.3%) | 303 (79.1%) |  | 255 (72.9%) | 253 (64.7%) |  |
| **Education** |  |  |  | <0.01 |  |  | <0.01 |  |  | <0.01 |  |  | <0.01 |
| ≤ High School | 135 (18.2%) | 97 (28.4%) | 38 (9.5%) |  | 98 (27.7%) | 37 (9.6%) |  | 92 (25.7%) | 43 (11.2%) |  | 98 (28.0%) | 37 (9.5%) |  |
| > High School | 606 (81.8%) | 244 (71.6%) | 362 (90.5%) |  | 256 (72.3%) | 350 (90.4%) |  | 266 (74.3%) | 340 (88.8%) |  | 252 (72.0%) | 354 (90.5%) |  |
| **Marital Status** |  |  |  | 0.20 |  |  | 0.33 |  |  | 0.65 |  |  | 0.71 |
| Married/ living as married | 328 (44.3%) | 142 (41.6%) | 186 (46.5%) |  | 150 (42.4%) | 178 (46.0%) |  | 155 (43.3%) | 173 (45.2%) |  | 152 (43.4%) | 176 (45.0%) |  |
| Not married | 413 (55.7%) | 199 (58.4%) | 214 (53.5%) |  | 204 (57.6%) | 209 (54.0%) |  | 203 (56.7%) | 210 (54.8%) |  | 198 (56.6%) | 215 (55.0%) |  |
| **Average census tract income** | 103,014 (48,533) | 100,406 (47,559) | 105,238 (49,299) | 0.17 | 97,922 (47,265) | 107,672 (49,267) | <0.01 | 100,971 (48,290) | 104,924 (48,745) | 0.26 | 99,127 (45,689) | 106,494 (50,751) | <0.01 |
| **Smoking status** |  |  |  | 0.08 |  |  | 0.03 |  |  | <0.01 |  |  | 0.42 |
| Never | 391 (52.8%) | 168 (49.3%) | 223 (55.8%) |  | 175 (49.4%) | 216 (55.8%) |  | 171 (47.8%) | 220 (57.4%) |  | 181 (51.7%) | 210 (53.7%) |  |
| Past | 307 (41.4%) | 156 (45.7%) | 151 (37.8%) |  | 163 (46.0%) | 144 (37.2%) |  | 173 (48.3%) | 134 (35.0%) |  | 152 (43.4%) | 155 (39.6%) |  |
| Current | 43 (5.8%) | 17 (5.0%) | 26 (6.5%) |  | 16 (4.5%) | 27 (7.0%) |  | 14 (3.9%) | 29 (7.6%) |  | 17 (4.9%) | 26 (6.6%) |  |
| **Alcohol consumption** |  |  |  | <0.01 |  |  | <0.01 |  |  | 0.01 |  |  | <0.01 |
| Never | 270 (36.4%) | 160 (46.9%) | 110 (27.5%) |  | 158 (44.6%) | 112 (28.9%) |  | 151 (42.2%) | 119 (31.1%) |  | 160 (45.7%) | 110 (28.1%) |  |
| Less than once a week | 237 (32.0.%) | 101 (29.6%) | 136 (34.0%) |  | 105 (29.7%) | 132 (34.1%) |  | 108 (30.2%) | 129 (33.7%) |  | 102 (29.1%) | 135 (34.5%) |  |
| 1 - 6 days per week | 208 (28.1%) | 73 (21.4%) | 135 (33.8%) |  | 83 (23.4%) | 125 (32.3%) |  | 89 (24.9%) | 119 (31.1%) |  | 81 (23.1%) | 127 (32.5%) |  |
| Every day | 26 (3.5%) | 7 (2.1%) | 19 (4.8%) |  | 8 (2.3%) | 18 (4.7%) |  | 10 (2.8%) | 16 (4.2%) |  | 7 (2.0%) | 19 (4.9%) |  |

Mean (SD) for continuous variables, N(%) categorical. ^1^Fishers exact test, Satterthwaite t-test. ^2^Cognitive measures are z-score standardized.^3^Total lead releases is a combination of air, land, water, and off-site lead releases reported by the facility to the Toxics Release Inventory. Off-site releases was not included in this analysis and therefore when combined, proportions of lead released through air, water, and land may not add to the total lead release number listed. Average census tract income rounded to the nearest whole number. Each cognitive outcome, dichotomized into “high” and “low” based on a cut-point of zero. lbs: pounds, km: kilometers, PM_2.5_: atmospheric particulate matter with a diameter < 2.5 micrometers.

**Supplemental Table 4.** Adjusted linear regression associations between residential distance to lead releasing facility and baseline cognition in the Kaiser Healthy Aging and Diverse Life Experiences Study (KHANDLE) analytic sample (n=1,638). Cognitive measures are z-score standardized.

| **Distance to lead**  **facility** | **Cognitive measure** | | | | | | | | | | | |
| --- | --- | --- | --- | --- | --- | --- | --- | --- | --- | --- | --- | --- |
|  | **Episodic memory** | | | **Semantic memory** | | | **Executive function** | | | **Global cognition** | | |
|  | **β** | **95% CI** | ***p***  **value** | **β** | **95% CI** | ***p* value** | **β** | **95% CI** | ***p* value** | **β** | **95% CI** | ***p* value** |
| **Minimally adjusted^1^** |  |  |  |  |  |  |  |  |  |  |  |  |
| Continuous (per 5 km) | -0.08 | (-0.05, -0.11) | <0.01 | -0.09 | (-0.06, -0.12) | <0.01 | -0.04 | (-0.01, -0.08) | <0.01 | -0.07 | (-0.05, -0.10) | <0.01 |
| Within 1.5 km | -0.21 | (-0.44, 0.02) | 0.07 | -0.34 | (-0.58, -0.10) | <0.01 | -0.44 | (-0.67, -0.20) | <0.01 | -0.33 | (-0.52, -0.14) | <0.01 |
| Within 3 km | -0.22 | (-0.33, -0.11) | <0.01 | -0.30 | (-0.41, -0.19) | <0.01 | -0.26 | (-0.37, -0.15) | <0.01 | -0.26 | (-0.35, -0.17) | <0.01 |
| Within 5 km | -0.25 | (-0.33, -0.16) | <0.01 | -0.27 | (-0.36, -0.18) | <0.01 | -0.16 | (-0.25, -0.07) | <0.01 | -0.23 | (-0.30, -0.15) | <0.01 |
| **Fully adjusted^2^** |  |  |  |  |  |  |  |  |  |  |  |  |
| Continuous (per 5 km) | -0.05 | (-0.02, -0.08) | <0.01 | -0.02 | (-0.05, 0.004) | 0.10 | 0.01 | (-0.02, 0.04) | 0.51 | -0.02 | (-0.04, 0.001) | 0.06 |
| Within 1.5 km | -0.09 | (-0.31, 0.13) | 0.43 | -0.16 | (-0.36, 0.03) | 0.11 | -0.24 | (-0.45, -0.04) | 0.02 | -0.17 | (-0.33, -0.01) | 0.04 |
| Within 3 km | -0.10 | (-0.21, 0.01) | 0.07 | -0.09 | (-0.19, 0.002) | 0.05 | -0.05 | (-0.15, 0.05) | 0.30 | -0.08 | (-0.16, -0.004) | 0.04 |
| Within 5 km | -0.15 | (-0.24, -0.06) | <0.01 | -0.07 | (-0.15, 0.01) | 0.07 | 0.002 | (-0.08, 0.08) | 0.99 | -0.07 | (-0.14, -0.01) | 0.02 |

^1^Minimally adjusted for baseline age and sex. **^2^** Fully adjusted for age, sex, education, census tract income, marital status, race/ethnicity, smoking status, and alcohol consumption. Continuous distance interpreted per every 5 km closer a residence is to a lead facility. β: beta value for linear regression estimate, km: kilometers.

**Supplemental Table 5.** Table of age coefficients from main regression analysis

|  | KHANDLE | | STAR | |
| --- | --- | --- | --- | --- |
|  | Beta for 1 year age increase | CI | Beta for 1 year age increase | CI |
| Episodic memory | -0.05 | (-0.05, -0.04) | -0.04 | (-0.05, -0.03) |
| Semantic memory | -0.04 | (-0.05, -0.04) | -0.03 | (-0.04, -0.03) |
| Executive function | -0.05 | (-0.05, -0.04) | -0.05 | (-0.06, -0.04) |
| Global cognition | -0.04 | (-0.05, -0.04) | -0.04 | (-0.05, -0.04) |

^1^Models adjusted for age, sex, education, census tract income, marital status, race/ethnicity, smoking status, and alcohol consumption.

**Supplemental Table 6.** Adjusted linear regression associations between residential distance to lead releasing facility and baseline cognition in the Study of Healthy Aging in African Americans (STAR) analytic sample (n=741). Cognitive measures are z-score standardized.

| **Distance to lead**  **facility** | **Cognitive measure** | | | | | | | | | | | |
| --- | --- | --- | --- | --- | --- | --- | --- | --- | --- | --- | --- | --- |
|  | **Episodic memory** | | | **Semantic memory** | | | **Executive function** | | | **Global cognition** | | |
|  | **β** | **95% CI** | **P value** | **β** | **95% CI** | **P value** | **β** | **95% CI** | **P value** | **β** | **95% CI** | **P value** |
| **Minimally adjusted^1^** |  |  |  |  |  |  |  |  |  |  |  |  |
| Continuous (per 5 km) | -0.08 | (-0.20, 0.03) | 0.15 | -0.12 | (-0.004, -0.24) | 0.04 | -0.10 | (-0.21, 0.01) | 0.07 | -0.10 | (-0.01, -0.19) | 0.03 |
| Within 1.5 km | -0.20 | (-0.40, -0.005) | 0.04 | -0.17 | (-0.38, 0.04) | 0.11 | -0.16 | (-0.35, 0.04) | 0.12 | -0.18 | (-0.33, -0.02) | 0.03 |
| Within 3 km | -0.11 | (-0.23, 0.02) | 0.09 | -0.14 | (-0.27, -0.01) | 0.04 | -0.17 | (-0.30, -0.05) | <0.01 | -0.14 | (-0.24, -0.04) | <0.01 |
| Within 5 km | -0.09 | (-0.26, 0.08) | 0.32 | -0.28 | (-0.46, -0.10) | <0.01 | -0.10 | (-0.27, 0.07) | 0.23 | -0.16 | (-0.29, -0.02) | 0.02 |
| **Fully adjusted^2^** |  |  |  |  |  |  |  |  |  |  |  |  |
| Continuous (per 5 km) | -0.03 | (-0.15, 0.08) | 0.59 | -0.07 | (-0.19, 0.05) | 0.25 | -0.02 | (-0.13, 0.09) | 0.67 | -0.04 | (-0.13, 0.05) | 0.35 |
| Within 1.5 km | -0.13 | (-0.33, 0.07) | 0.19 | -0.08 | (-0.28, 0.13) | 0.46 | -0.05 | (-0.23, 0.14) | 0.64 | -0.08 | (-0.23, 0.07) | 0.28 |
| Within 3 km | -0.06 | (-0.19, 0.07) | 0.37 | -0.10 | (-0.24, 0.04) | 0.16 | -0.09 | (-0.22, 0.04) | 0.19 | -0.08 | (-0.18, 0.02) | 0.12 |
| Within 5 km | 0.02 | (-0.16, 0.21) | 0.79 | -0.20 | (-0.39, -0.02) | 0.03 | 0.08 | (-0.10, 0.26) | 0.37 | -0.03 | (-0.17, 0.11) | 0.64 |

^1^Minimally adjusted for baseline age and sex. ^2^Fully adjusted for age, sex, education, census tract income, marital status, race/ethnicity, smoking status, and alcohol consumption. Continuous distance interpreted per every 5 km closer a residence is to a lead facility. β: beta value for linear regression estimate, km: kilometers.

**Supplemental Table 7.** Table of mean difference and 95% confidence interval for meta-analysis across the KHANDLE and STAR analytic sample cohorts.

| **Distance to lead**  **facility** | **Cognitive measure** | | | | | | | | | | | |
| --- | --- | --- | --- | --- | --- | --- | --- | --- | --- | --- | --- | --- |
|  | **Episodic memory** | | | **Semantic memory** | | | **Executive function** | | | **Global cognition** | | |
|  | **MD** | **95% CI** | **P value** | **MD** | **95% CI** | **P value** | **MD** | **95% CI** | **P value** | **MD** | **95% CI** | **P value** |
| **Minimally adjusted^1^** |  |  |  |  |  |  |  |  |  |  |  |  |
| Continuous (per 5 km) | -0.08 | (-0.11, -0.05) | <0.01 | -0.09 | (-0.12, -0.06) | <0.01 | -0.05 | (-0.08, -0.02) | <0.01 | -0.07 | (-0.10, -0.05) | <0.01 |
| Within 1.5 km | -0.21 | (-0.36, -0.06) | <0.01 | -0.24 | (-0.41, -0.08) | <0.01 | -0.29 | (-0.56,-0.01) | 0.04 | -0.24 | (-0.39,-0.09) | <0.01 |
| Within 3 km | -0.17 | (-0.28, -0.06) | <0.01 | -0.23 | (-0.38, -0.07) | <0.01 | -0.22 | (-0.30, -0.14) | <0.01 | -0.20 | (-0.32, -0.09) | <0.01 |
| Within 5 km | -0.18 | (-0.34, -0.03) | 0.02 | -0.27 | (-0.35, -0.19) | <0.01 | -0.15 | (-0.23, -0.07) | <0.01 | -0.21 | (-0.27, -0.15) | <0.01 |
| **Fully adjusted^2^** |  |  |  |  |  |  |  |  |  |  |  |  |
| Continuous (per 5 km) | -0.05 | (-0.08, -0.02) | <0.01 | -0.03 | (-0.05, -0.001) | 0.06 | 0.008 | (-0.02, 0.04) | 0.60 | -0.02 | (-0.04, -0.0008) | 0.04 |
| Within 1.5 km | -0.11 | (-0.26, 0.03) | 0.13 | -0.12 | (-0.26, 0.02) | 0.10 | -0.14 | (-0.33, 0.05) | 0.16 | -0.12 | (-0.23,-0.01) | 0.03 |
| Within 3 km | -0.08 | (-0.17, 0.0001) | 0.05 | -0.10 | (-0.18, -0.02) | 0.02 | -0.06 | (-0.15, 0.01) | 0.10 | -0.08 | (-0.14, -0.02) | <0.01 |
| Within 5 km | -0.08 | (-0.25, 0.08) | 0.33 | -0.11 | (-0.23, 0.01) | 0.06 | 0.02 | (-0.06,0.09) | 0.68 | -0.07 | (-0.12, -0.009) | 0.02 |

^1^Minimally adjusted for age and sex. ^2^Fully adjusted for age, sex, education, census tract income, marital status, race/ethnicity, smoking status, alcohol consumption. KHANDLE: Kaiser Healthy Aging and Diverse Life Experiences Study; STAR: Study of Healthy Aging in African Americans, MD: mean difference.

**Supplemental Table 8.** Descriptive statistics for the complete case sensitivity analysis included and excluded participants in the KHANDLE and STAR cohorts.

|  | **Cohort** | | | | | | | |
| --- | --- | --- | --- | --- | --- | --- | --- | --- |
|  |  | **KHANDLE** |  |  |  | **STAR** |  |  |
| **Characteristic** | **Overall,**  N = 1,663 | **Excluded,**  N = 73 | **Included,**  N = 1,590 | ***p* value*^1^*** | **Overall,**  N = 746 | **Excluded,**  N = 25 | **Included,**  N = 721 | ***p* value*^1^*** |
| **Global cognition^2^** | 0.01 (0.81) | -0.22 (0.91) | 0.02 (0.81) | 0.07 | 0.01 (0.81) | -0.51 (0.90) | 0.03 (0.80) | 0.02 |
| (missing) | 1 | 1 | - |  | - | - | - |  |
| **Executive function^2^** | 0.01 (1.00) | -0.38 (0.99) | 0.02 (0.99) | <0.01 | 0.01 (0.99) | -0.73 (.99) | 0.03 (0.99) | <0.01 |
| (missing) | 10 | 10 | - |  | - | - | - |  |
| **Episodic memory^2^** | 0.01 (1.00) | -0.08 (1.20) | 0.01 (0.99) | 0.60 | 0.02 (0.99) | -0.37 (1.00) | 0.03 (0.99) | 0.08 |
| (missing) | 4 | 4 | - |  | 2 | 2 | - |  |
| **Semantic memory^2^** | 0.01 (1.00) | -0.27 (1.10) | 0.02 (0.99) | 0.06 | 0.00 (0.99) | -0.50 (1.10) | 0.02 (0.98) | 0.03 |
| (missing) | 7 | 7 | - |  | 3 | 3 | - |  |
| **Average distance to facility (km)** | 8.2 (6.9) | 7.5 (6.4) | 8.2 (6.9) | 0.36 | 3.6 (2.8) | 3.4 (1.1) | 3.6 (2.8) | 0.40 |
| **Lead releasing facility within 1.5km** |  |  |  | 0.19 |  |  |  | 0.34 |
| No | 1602 (96.3%) | 68 (93.2%) | 1,534 (96.5%) |  | 661 (88.6%) | 22 (95.7%) | 637 (88.3%) |  |
| Yes | 61 (3.7%) | 5 (6.8%) | 56 (3.5%) |  | 85 (11.4%) | 1 (4.3%) | 84 (11.7%) |  |
| **Lead releasing facility within 3km** |  |  |  | 0.46 |  |  |  | 0.16 |
| No | 1331 (80.0%) | 56 (76.7%) | 1,275 (80.2%) |  | 401 (53.8%) | 15 (65.2%) | 384 (53.3%) |  |
| Yes | 332 (20.0%) | 17 (23.3%) | 315 (19.8%) |  | 345 (46.2%) | 8 (34.8%) | 337 (46.7%) |  |
| **Lead releasing facility within 5km** |  |  |  | 0.23 |  |  |  | 0.16 |
| No | 907 (54.5%) | 37 (50.7%) | 870 (54.7%) |  | 122 (16.4%) | - | 66 (9.2%) |  |
| Yes | 756 (45.5%) | 45 (61.6%) | 862 (54.2%) |  | 624 (83.6%) | 23 (100.0%) | 655 (90.8%) |  |
| **Total lead releases (lbs)^3^** | 2,307.5 (5,331.9) | 2,379.1 (4,899.1) | 2,304.2 (5,352.3) | 0.90 | 4,956.1 (6,245.5) | 3,516.3 (5,831.1) | 5,006.1 (6,257.2) | 0.20 |
| **Air lead releases (lbs)** | 11.3 (30.1) | 15.5 (35.2) | 11.1 (29.9) | 0.30 | 27.0 (37.0) | 19.8 (34.2) | 27.2 (37.1) | 0.30 |
| **Water lead releases (lbs)** | 1.5 (7.2) | 2.2 (9.9) | 1.5 (7.0) | 0.56 | 0.8 (3.3) | 0.2 (0.7) | 0.8 (3.3) | <0.01 |
| **Land lead releases (lbs)** | 10.2 (230.7) | 0.0 | 10.7 (236.0) | 0.07 | 0.2 (4.6) | 0.0 (0.0) | 0.2 (4.7) | 0.30 |
| **Age at interview** | 76.1 (7.2) | 78.7 (8.8) | 76 (7.1) | 0.01 | 68.8 (8.8) | 69.7 (7.8) | 68.7 (8.8) | 0.50 |
| **Sex** |  |  |  | 0.90 |  |  |  | 0.20 |
| Male | 684 (41.1%) | 29 (39.7%) | 655 (41.2%) |  | 235 (31.5%) | 11 (47.8%) | 224 (31.1%) |  |
| Female | 979 (58.9%) | 44 (60.3%) | 935 (58.8%) |  | 511 (68.5%) | 12 (52.2%) | 497 (68.9%) |  |
| **Race/ethnicity** |  |  |  | <0.01 |  |  |  | <0.01 |
| Asian | 412 (24.8%) | 15 (20.5%) | 397 (25.0%) |  | - | - | - |  |
| Black | 431 (25.9%) | 23 (31.5%) | 408 (25.7%) |  | 736 (98.7%) | 19 (82.6%) | 715 (99.2%) |  |
| LatinX | 327 (19.7%) | 13 (17.8%) | 314 (19.7%) |  | 6 (0.8%) | - | 6 (0.8%) |  |
| Native American | 3 (0.2%) | 3 (4.1%) | - |  | 4 (0.5%) | 4 (17.4%) | - |  |
| White | 490 (29.5%) | 19 (26.0%) | 471 (29.6%) |  | - | - | - |  |
| (missing) | 1 | 1 | - |  |  |  |  |  |
| **Education** |  |  |  | <0.01 |  |  |  | <0.01 |
| ≤ High School | 281 (16.9%) | 18 (24.7%) | 262 (16.5%) |  | 136 (18.2%) | 7 (30.4%) | 128 (17.8%) |  |
| > High School | 1382 (83.1%) | 54 (74.0%) | 1,328 (83.5%) |  | 610 (81.8%) | 13 (56.5%) | 593 (82.2%) |  |
| (missing) | 1 | 1 | - |  | 3 | 3 | - |  |
| **Marital Status** |  |  |  | <0.01 |  |  |  | <0.01 |
| Married/ living as married | 944 (56.8%) | 25 (34.2%) | 908 (57.1%) |  | 329 (44.1%) | 1 (4.3%) | 320 (44.4%) |  |
| Not Married | 714 (42.9%) | 16 (21.9%) | 682 (42.9%) |  | 416 (55.8%) | 5 (21.7%) | 401 (55.6%) |  |
| (missing) | 26 | 26 | - |  | 17 | 17 | - |  |
| **Average census tract income** | 1,213,78 (52,401) | 118,853 (52,807) | 121,502 (52,344) | 0.68 | 103,178 (48,673) | 100,955 (45,476) | 103,255 (48,808) | 0.80 |
| (missing) | 3 | 3 | - |  | - | - | - |  |
| **Smoking status** |  |  |  | <0.01 |  |  |  | 0.74 |
| Never | 920 (55.3%) | 32 (43.8%) | 887 (55.8%) |  | 396 (53.1%) | 13 (56.5%) | 382 (53.0%) |  |
| Former | 691 (41.6%) | 31 (42.5%) | 656 (41.3%) |  | 307 (41.2%) | 8 (34.8%) | 298 (41.3%) |  |
| Current | 52 (3.1%) | 4 (5.5%) | 47 (3.0%) |  | 43 (5.8%) | 2 (8.7%) | 41 (5.7%) |  |
| (missing) | 6 | 6 | - |  | - | - | - |  |
| **Alcohol consumption** |  |  |  | <0.01 |  |  |  | <0.01 |
| Never | 492 (29.6%) | 24 (32.9%) | 466 (29.3%) |  | 274 (36.7%) | 15 (65.2%) | 259 (35.9%) |  |
| Less than once a week | 580 (34.9%) | 21 (28.8%) | 551 (34.7%) |  | 237 (31.8%) | 3 (13.0%) | 232 (32.2%) |  |
| 1-6 days per week | 430 (25.9%) | 14 (19.2%) | 414 (26.0%) |  | 208 (27.9%) | 4 (17.4%) | 204 (28.3%) |  |
| Every day | 161 (9.7%) | 1 (1.4%) | 159 (10.0%) |  | 27 (3.6%) | 1 (4.3%) | 26 (3.6%) |  |
| (missing) | 12 | 12 | - |  | - | - | - |  |

Mean (SD) for continuous variables, N(%) categorical. ^1^Fishers exact test, Satterthwaite t-test. ^2^Cognitive measures are z-score standardized. ^3^Total lead releases is a combination of air, land, water, and off-site lead releases reported by the facility to the Toxics Release Inventory. Off-site releases was not included in this analysis and therefore when combined, proportions of lead released through air, water, and land may not add to the total lead release number listed. Average census tract income rounded to the nearest whole number, KHANDLE: Kaiser Healthy Aging and Diverse Life Experiences Study, STAR: Study of Healthy Aging in African Americans, lbs: pounds, km: kilometers.

**Supplemental Table 9**. Sensitivity results for complete case analysis adjusted linear regression associations between residential distance to lead releasing facility and baseline cognition in KHANDLE analytic sample (n=1,590). Cognitive measures are z-score standardized.

| **Distance to lead**  **facility** | **Cognitive measure** | | | | | | | | | | | |
| --- | --- | --- | --- | --- | --- | --- | --- | --- | --- | --- | --- | --- |
|  | **Episodic memory** | | | **Semantic memory** | | | **Executive function** | | | **Global cognition** | | |
|  | **β** | **95% CI** | **P value** | **β** | **95% CI** | **P value** | **β** | **95% CI** | **P value** | **β** | **95% CI** | **P value** |
| **Minimally adjusted^1^** |  |  |  |  |  |  |  |  |  |  |  |  |
| Continuous (per 5 km) | -0.07 | (-0.04, -0.10) | <0.01 | -0.09 | (-0.05, -0.12) | <0.01 | -0.04 | (-0.01, -0.07) | 0.01 | -0.07 | (-0.04, -0.09) | <0.01 |
| Within 1.5 km | -0.16 | (-0.40, 0.08) | 0.19 | -0.31 | (-0.56, -0.07) | 0.01 | -0.38 | (-0.63, -0.14) | <0.01 | -0.28 | (-0.48, -0.09) | <0.01 |
| Within 3 km | -0.20 | (-0.31, -0.10) | <0.01 | -0.28 | (-0.40, -0.17) | <0.01 | -0.25 | (-0.36, -0.13) | <0.01 | -0.25 | (-0.34, -0.16) | <0.01 |
| Within 5 km | -0.22 | (-0.31, -0.14) | <0.01 | -0.26 | (-0.35, -0.17) | <0.01 | -0.16 | (-0.25, -0.07) | <0.01 | -0.21 | (-0.29, -0.14) | <0.01 |
| **Fully adjusted^2^** |  |  |  |  |  |  |  |  |  |  |  |  |
| Continuous (per 5 km) | -0.04 | (-0.01, -0.08) | 0.01 | -0.02 | (-0.04, 0.01) | 0.17 | 0.01 | (-0.02, 0.04) | 0.45 | -0.02 | (-0.04, 0.01) | 0.13 |
| Within 1.5 km | -0.05 | (-0.28, 0.18) | 0.69 | -0.17 | (-0.37, 0.04) | 0.11 | -0.21 | (-0.43, 0.002) | 0.05 | -0.14 | (-0.31, 0.02) | 0.09 |
| Within 3 km | -0.09 | (-0.2, 0.02) | 0.11 | -0.08 | (-0.18, 0.02) | 0.11 | -0.05 | (-0.15, 0.06) | 0.38 | -0.07 | (-0.15, 0.01) | 0.08 |
| Within 5 km | -0.13 | (-0.22, -0.05) | <0.01 | -0.07 | (-0.15, 0.01) | 0.09 | <0.01 | (-0.08, 0.09) | 0.94 | -0.07 | (-0.13, -0.002) | 0.04 |

^1^Minimally adjusted for age and sex. ^2^Fully adjusted for age, sex, education, census tract income, marital status, race/ethnicity, smoking status, alcohol consumption. Complete case: non-missing values for all observations required for study inclusion. Continuous distance interpreted per every 5 km closer a residence is to a lead facility. β: beta value for linear regression estimate, km: kilometers, KHANDLE: Kaiser Healthy Aging and Diverse Life Experiences Study.

**Supplemental Table 10**. Sensitivity results for complete case analysis adjusted linear regression associations between residential distance to lead releasing facility and baseline cognition in STAR analytic sample (n=721). Cognitive measures are z-score standardized.

| **Distance to lead**  **facility** | **Cognitive measure** | | | | | | | | | | | |
| --- | --- | --- | --- | --- | --- | --- | --- | --- | --- | --- | --- | --- |
|  | **Episodic memory** | | | **Semantic memory** | | | **Executive function** | | | **Global cognition** | | |
|  | **β** | **95% CI** | **P value** | **β** | **95% CI** | **P value** | **β** | **95% CI** | **P value** | **β** | **95% CI** | **P value** |
| **Minimally adjusted^1^** |  |  |  |  |  |  |  |  |  |  |  |  |
| Continuous (per 5 km) | -0.08 | (-0.19, 0.03) | 0.16 | -0.13 | (-0.01, -0.25) | 0.03 | -0.10 | (-0.21, 0.01) | 0.07 | -0.10 | (-0.02, -0.19) | 0.02 |
| Within 1.5 km | -0.21 | (-0.41, -0.01) | 0.04 | -0.19 | (-0.39, 0.02) | 0.07 | -0.17 | (-0.37, 0.02) | 0.08 | -0.19 | (-0.35, -0.04) | 0.02 |
| Within 3 km | -0.12 | (-0.25, 0.01) | 0.06 | -0.18 | (-0.31, -0.05) | 0.01 | -0.19 | (-0.32, -0.07) | <0.01 | -0.16 | (-0.26, -0.07) | <0.01 |
| Within 5 km | -0.08 | (-0.25, 0.09) | 0.38 | -0.26 | (-0.44, -0.09) | <0.01 | -0.08 | (-0.25, 0.08) | 0.33 | -0.14 | (-0.28, -0.01) | 0.04 |
| **Fully adjusted^2^** |  |  |  |  |  |  |  |  |  |  |  |  |
| Continuous (per 5 km) | -0.03 | (-0.14, 0.08) | 0.6 | -0.08 | (-0.20, 0.04) | 0.19 | 0.02 | (-0.13, 0.09) | 0.66 | -0.04 | (-0.13, 0.04) | 0.32 |
| Within 1.5 km | -0.14 | (-0.33, 0.06) | 0.17 | -0.09 | (-0.30, 0.11) | 0.36 | -0.06 | (-0.25, 0.13) | 0.52 | -0.10 | (-0.25, 0.05) | 0.20 |
| Within 3 km | -0.07 | (-0.20, 0.07) | 0.34 | -0.14 | (-0.28, 0.003) | 0.05 | -0.10 | (-0.23, 0.03) | 0.13 | -0.10 | (-0.21, 0.002) | 0.05 |
| Within 5 km | 0.03 | (-0.15, 0.22) | 0.72 | -0.19 | (-0.38, -0.004) | 0.05 | 0.10 | (-0.07, 0.28) | 0.26 | -0.02 | (-0.16, 0.12) | 0.79 |

^1^Minimally adjusted for age and sex. ^2^Fully adjusted for age, sex, education, census tract income, marital status, race/ethnicity, smoking status, alcohol consumption. Complete case: non-missing values for all observations required for study inclusion. Continuous distance interpreted per every 5 km closer a residence is to a lead facility. β: beta value for linear regression estimate, km: kilometers, STAR: Study of Healthy Aging in African Americans.

**Supplemental Table 11:** Sensitivity results for age stratified analysis adjusted linear regression associations between residential distance to lead releasing facility and baseline cognition in KHANDLE analytic sample (n=1,638). Cognitive measures are z-score standardized.

| **Distance to lead**  **facility** | **Age** | **Cognitive measure** | | | | | | | | | | | |
| --- | --- | --- | --- | --- | --- | --- | --- | --- | --- | --- | --- | --- | --- |
|  |  | **Episodic memory** | | | **Semantic memory** | | | **Executive function** | | | **Global cognition** | | |
|  |  | **β** | **95% CI** | **P value** | **β** | **95% CI** | **P value** | **β** | **95% CI** | **P value** | **β** | **95% CI** | **P value** |
| **Minimally adjusted**^1^ |  |  |  |  |  |  |  |  |  |  |  |  |  |
| Continuous (per 5 km) | < 70 | -0.09 | (-0.01, -0.16) | 0.03 | -0.10 | (-0.02, -0.17) | 0.01 | -0.07 | (-0.14, 0.01) | 0.08 | -0.08 | (-0.02, -0.15) | 0.01 |
|  | 70-74 | -0.10 | (-0.05, -0.16) | <0.01 | -0.04 | (-0.02, 0.11) | 0.17 | -0.04 | (-0.10, 0.02) | 0.24 | 0.06 | (0.01, 0.11) | 0.01 |
|  | 75-79 | -0.05 | (-0.11, 0.01) | 0.12 | -0.12 | (0.05, 0.19) | <0.01 | -0.05 | (-0.12, 0.02) | 0.15 | 0.07 | (0.02, 0.13) | 0.01 |
|  | ≥ 80 | -0.08 | (-0.02, -0.13) | 0.01 | -0.10 | (0.04, 0.16) | <0.01 | -0.03 | (-0.09, 0.03) | 0.29 | 0.07 | (0.02, 0.12) | <0.01 |
| Within 1.5 km | < 70 | -0.05 | (-0.53, 0.43) | 0.84 | -0.05 | (-0.51, 0.41) | 0.83 | -0.17 | (-0.63, 0.30) | 0.48 | -0.09 | (-0.47, 0.29) | 0.64 |
|  | 70-74 | 0.07 | (-0.36, 0.49) | 0.75 | -0.62 | (-1.08, -0.17) | 0.01 | -0.32 | (-0.77, 0.14) | 0.17 | -0.29 | (-0.65, 0.07) | 0.11 |
|  | 75-79 | -0.50 | (-0.91, -0.09) | 0.02 | -0.50 | (-0.93, -0.06) | 0.02 | -0.79 | (-1.22, -0.36) | <0.01 | -0.60 | (-0.93, -0.26) | <0.01 |
|  | ≥ 80 | -0.47 | (-1.07, 0.12) | 0.12 | -0.07 | (-0.70, 0.56) | 0.83 | -0.57 | (-1.18, 0.04) | 0.07 | -0.37 | (-0.87, 0.13) | 0.14 |
| Within 3 km | < 70 | -0.07 | (-0.30, 0.15) | 0.53 | -0.07 | (-0.29, 0.14) | 0.51 | -0.07 | (-0.29, 0.15) | 0.51 | -0.07 | (-0.25, 0.10) | 0.42 |
|  | 70-74 | -0.32 | (-0.52, -0.12) | <0.01 | -0.29 | (-0.51, -0.08) | 0.01 | -0.23 | (-0.44, -0.02) | 0.03 | -0.28 | (-0.45, -0.12) | <0.01 |
|  | 75-79 | -0.23 | (-0.45, 0.004) | 0.05 | -0.38 | (-0.62, -0.14) | <0.01 | -0.31 | (-0.55, -0.07) | 0.01 | -0.31 | (-0.49, -0.12) | <0.01 |
|  | ≥ 80 | -0.33 | (-0.55, -0.11) | <0.01 | -0.54 | (-0.77, -0.31) | <0.01 | -0.52 | (-0.74, -0.29) | <0.01 | -0.46 | (-0.64, -0.28) | <0.01 |
| Within 5 km | < 70 | -0.12 | (-0.31, 0.06) | 0.19 | -0.28 | (-0.45, -0.10) | <0.01 | -0.11 | (-0.29, 0.08) | 0.26 | -0.17 | (-0.31, -0.02) | 0.02 |
|  | 70-74 | -0.32 | (-0.47, -0.16) | <0.01 | -0.04 | (-0.22, 0.13) | 0.61 | -0.11 | (-0.28, 0.06) | 0.19 | -0.16 | (-0.29, -0.02) | 0.02 |
|  | 75-79 | -0.17 | (-0.36, 0.03) | 0.09 | -0.48 | (-0.68, -0.28) | <0.01 | -0.29 | (-0.49, -0.08) | 0.01 | -0.31 | (-0.47, -0.15) | <0.01 |
|  | ≥ 80 | -0.35 | (-0.51, -0.18) | <0.01 | -0.35 | (-0.53, -0.17) | <0.01 | -0.20 | (-0.37, -0.03) | 0.02 | -0.30 | (-0.44, -0.16) | <0.01 |
| **Fully adjusted^2^** |  |  |  |  |  |  |  |  |  |  |  |  |  |
| Continuous (per 5 km) | < 70 | -0.04 | (-0.12, 0.03) | 0.25 | -0.03 | (-0.10, 0.03) | 0.28 | -0.03 | (-0.09, 0.04) | 0.44 | -0.04 | (-0.09, 0.02) | 0.19 |
|  | 70-74 | -0.07 | (-0.02, -0.13) | 0.01 | 0.01 | (-0.05, 0.06) | 0.75 | 0.01 | (-0.04, 0.07) | 0.65 | -0.02 | (-0.06, 0.03) | 0.44 |
|  | 75-79 | -0.03 | (-0.09, 0.03) | 0.36 | -0.04 | (-0.09, 0.02) | 0.18 | 0.02 | (-0.04, 0.08) | 0.53 | -0.02 | (-0.06, 0.03) | 0.47 |
|  | ≥ 80 | -0.05 | (-0.11, 0.01) | 0.09 | -0.03 | (-0.08, 0.02) | 0.25 | 0.02 | (-0.03, 0.08) | 0.39 | -0.02 | (-0.06, 0.02) | 0.39 |
| Within 1.5 km | < 70 | 0.01 | (-0.44, 0.46) | 0.95 | 0.19 | (-0.18, 0.55) | 0.31 | 0.02 | (-0.38, 0.42) | 0.93 | 0.07 | (-0.24, 0.38) | 0.64 |
|  | 70-74 | 0.29 | (-0.11, 0.69) | 0.16 | -0.37 | (-0.76, 0.01) | 0.06 | -0.05 | (-0.44, 0.35) | 0.82 | -0.04 | (-0.35, 0.26) | 0.78 |
|  | 75-79 | -0.38 | (-0.78, 0.02) | 0.06 | -0.26 | (-0.61, 0.09) | 0.15 | -0.50 | (-0.86, -0.14) | 0.01 | -0.38 | (-0.66, -0.10) | 0.01 |
|  | ≥ 80 | -0.31 | (-0.89, 0.28) | 0.30 | -0.003 | (-0.53, 0.52) | 0.99 | -0.47 | (-1.01, 0.08) | 0.10 | -0.26 | (-0.69, 0.18) | 0.25 |
| Within 3 km | < 70 | -0.03 | (-0.25, 0.19) | 0.81 | 0.06 | (-0.12, 0.23) | 0.54 | 0.05 | (-0.15, 0.24) | 0.64 | 0.03 | (-0.13, 0.18) | 0.74 |
|  | 70-74 | -0.18 | (-0.38, 0.01) | 0.07 | -0.05 | (-0.24, 0.14) | 0.60 | 0.01 | (-0.18, 0.21) | 0.89 | -0.07 | (-0.22, 0.08) | 0.34 |
|  | 75-79 | -0.05 | (-0.29, 0.19) | 0.68 | -0.04 | (-0.25, 0.17) | 0.70 | 0.02 | (-0.19, 0.23) | 0.86 | -0.02 | (-0.19, 0.14) | 0.78 |
|  | ≥ 80 | -0.18 | (-0.40, 0.05) | 0.12 | -0.33 | (-0.53, -0.12) | <0.01 | -0.32 | (-0.53, -0.10) | <0.01 | -0.27 | (-0.44, -0.11) | <0.01 |
| Within 5 km | < 70 | -0.06 | (-0.24, 0.12) | 0.49 | -0.16 | (-0.31, -0.02) | 0.03 | -0.04 | (-0.20, 0.12) | 0.64 | -0.09 | (-0.21, 0.03) | 0.16 |
|  | 70-74 | -0.23 | (-0.39, -0.08) | <0.01 | 0.04 | (-0.11, 0.19) | 0.56 | -0.03 | (-0.19, 0.12) | 0.66 | -0.07 | (-0.19, 0.04) | 0.22 |
|  | 75-79 | -0.08 | (-0.28, 0.13) | 0.46 | -0.13 | (-0.31, 0.05) | 0.16 | 0.02 | (-0.16, 0.20) | 0.84 | -0.06 | (-0.21, 0.08) | 0.40 |
|  | ≥ 80 | -0.22 | (-0.40, -0.04) | 0.02 | -0.09 | (-0.25, 0.07) | 0.29 | 0.04 | (-0.13, 0.21) | 0.67 | -0.09 | (-0.22, 0.04) | 0.19 |

^1^Minimally adjusted for baseline age and sex. ^2^Fully adjusted for age, sex, education, census tract income, marital status, race/ethnicity, smoking status, alcohol consumption. Continuous distance interpreted per every 5 km closer a residence is to a lead facility. β: beta value for linear regression estimate, km: kilometers, KHANDLE: Kaiser Healthy Aging and Diverse Life Experiences Study

**Supplemental Table 12:** Sensitivity results for age stratified adjusted linear regression associations between residential distance to lead releasing facility and baseline cognition in STAR analytic sample (n=741). Cognitive measures are z-score standardized.

| **Distance to lead**  **facility** | **Age** | **Cognitive measure** | | | | | | | | | | | |
| --- | --- | --- | --- | --- | --- | --- | --- | --- | --- | --- | --- | --- | --- |
|  |  | **Episodic memory** | | | **Semantic memory** | | | **Executive function** | | | **Global cognition** | | |
|  |  | **β** | **95% CI** | **P value** | **β** | **95% CI** | **P value** | **β** | **95% CI** | **P value** | **β** | **95% CI** | **P value** |
| **Minimally adjusted** ^1^ |  |  |  |  |  |  |  |  |  |  |  |  |  |
| Continuous (per 5 km) | < 70 | -0.04 | (-0.16, 0.08) | 0.54 | -0.07 | (-0.19, 0.05) | 0.23 | -0.05 | (-0.17, 0.07) | 0.38 | -0.06 | (-0.15, 0.04) | 0.25 |
|  | 70-74 | 0.003 | (-0.40, 0.41) | 0.99 | -0.13 | (-0.53, 0.26) | 0.51 | -0.22 | (-0.60, 0.16) | 0.26 | -0.12 | (-0.42, 0.19) | 0.46 |
|  | 75-79 | -0.91 | (-0.42, -1.40) | <0.01 | -0.43 | (-1.10, 0.22) | 0.20 | -0.46 | (-0.98, 0.05) | 0.08 | -0.60 | (-0.17, -1.03) | <0.01 |
|  | ≥ 80 | -0.32 | (-0.88, 0.25) | 0.27 | -0.83 | (-0.23, -1.40) | <0.01 | -0.78 | (-0.19, -1.37) | 0.01 | -0.64 | (-0.15, -1.10) | 0.01 |
| Within 1.5 km | < 70 | -0.07 | (-0.33, 0.20) | 0.62 | -0.09 | (-0.35, 0.18) | 0.52 | 0.04 | (-0.23, 0.30) | 0.79 | -0.04 | (-0.24, 0.16) | 0.70 |
|  | 70-74 | -0.43 | (-0.88, 0.02) | 0.06 | 0.26 | (-0.18, 0.71) | 0.25 | -0.46 | (-0.88, -0.03) | 0.04 | -0.21 | (-0.56, 0.14) | 0.23 |
|  | 75-79 | -0.25 | (-0.86, 0.36) | 0.41 | -0.45 | (-1.21, 0.30) | 0.24 | -0.20 | (-0.81, 0.40) | 0.51 | -0.30 | (-0.82, 0.21) | 0.25 |
|  | ≥ 80 | -0.36 | (-0.88, 0.16) | 0.17 | -0.92 | (-1.47, -0.37) | <0.01 | -0.56 | (-1.12, 0.0007) | 0.05 | -0.61 | (-1.07, -0.16) | 0.01 |
| Within 3 km | < 70 | -0.09 | (-0.25, 0.07) | 0.29 | -0.17 | (-0.32, -0.01) | 0.04 | -0.08 | (-0.24, 0.08) | 0.34 | -0.11 | (-0.23, 0.01) | 0.08 |
|  | 70-74 | -0.11 | (-0.44, 0.23) | 0.53 | 0.02 | (-0.30, 0.35) | 0.89 | -0.34 | (-0.65, -0.03) | 0.03 | -0.14 | (-0.39, 0.11) | 0.27 |
|  | 75-79 | -0.31 | (-0.69, 0.07) | 0.11 | -0.01 | (-0.49, 0.47) | 0.96 | -0.33 | (-0.70, 0.05) | 0.09 | -0.22 | (-0.54, 0.11) | 0.19 |
|  | ≥ 80 | -0.23 | (-0.57, 0.11) | 0.19 | -0.56 | (-0.93, -0.20) | <0.01 | -0.60 | (-0.96, -0.25) | <0.01 | -0.46 | (-0.76, -0.17) | <0.01 |
| Within 5 km | < 70 | -0.05 | (-0.25, 0.15) | 0.62 | -0.26 | (-0.45, -0.06) | 0.01 | -0.09 | (-0.29, 0.11) | 0.39 | -0.13 | (-0.29, 0.02) | 0.09 |
|  | 70-74 | 0.29 | (-0.25, 0.82) | 0.29 | -0.17 | (-0.69, 0.36) | 0.53 | -0.001 | (-0.51, 0.51) | 0.90 | 0.04 | (-0.37, 0.45) | 0.85 |
|  | 75-79 | -0.78 | (-1.26, -0.31) | <0.01 | -0.72 | (-1.33, -0.11) | 0.02 | -0.46 | (-0.95, 0.03) | 0.06 | -0.65 | (-1.06, -0.25) | <0.01 |
|  | ≥ 80 | -0.03 | (-0.60, 0.53) | 0.91 | -0.19 | (-0.82, 0.43) | 0.55 | -0.09 | (-0.70, 0.53) | 0.78 | -0.10 | (-0.61, 0.40) | 0.69 |
| **Fully adjusted^2^** |  |  |  |  |  |  |  |  |  |  |  |  |  |
| Continuous (per 5 km) | < 70 | 0.02 | (-0.11, 0.14) | 0.78 | 0.05 | (-0.07, 0.17) | 0.37 | 0.02 | (-0.11, 0.14) | 0.80 | -0.03 | (-0.12, 0.06) | 0.54 |
|  | 70-74 | -0.10 | (-0.51, 0.31) | 0.63 | 0.003 | (-0.39, 0.39) | 0.99 | 0.08 | (-0.32, 0.47) | 0.70 | 0.007 | (-0.30, 0.31) | 0.97 |
|  | 75-79 | 0.50 | (-0.12, 1.20) | 0.11 | 0.90 | (-0.76, 0.93) | 0.84 | -0.10 | (-0.73, 0.53) | 0.76 | -0.16 | (-0.68, 0.36) | 0.54 |
|  | ≥ 80 | 0.26 | (-0.41, 0.92) | 0.45 | 0.69 | (-0.01, 1.40) | 0.05 | 0.53 | (-0.15, 1.20) | 0.13 | -0.49 | (-1.10, 0.08) | 0.09 |
| Within 1.5 km | < 70 | -0.04 | (-0.31, 0.22) | 0.75 | -0.04 | (-0.30, 0.21) | 0.74 | 0.10 | (-0.16, 0.36) | 0.45 | 0.004 | (-0.20, 0.20) | 0.97 |
|  | 70-74 | -0.34 | (-0.79, 0.10) | 0.13 | 0.41 | (-0.01, 0.83) | 0.06 | -0.39 | (-0.81, 0.03) | 0.07 | -0.11 | (-0.43, 0.22) | 0.51 |
|  | 75-79 | -0.005 | (-0.57, 0.56) | 0.99 | -0.38 | (-1.14, 0.37) | 0.32 | 0.08 | (-0.49, 0.65) | 0.79 | -0.10 | (-0.57, 0.37) | 0.67 |
|  | ≥ 80 | -0.24 | (-0.75, 0.26) | 0.35 | -0.78 | (-1.31, -0.25) | <0.01 | -0.33 | (-0.85, 0.19) | 0.22 | -0.45 | (-0.88, -0.02) | 0.04 |
| Within 3 km | < 70 | -0.07 | (-0.24, 0.10) | 0.42 | -0.17 | (-0.34, -0.01) | 0.04 | -0.02 | (-0.18, 0.15) | 0.84 | -0.09 | (-0.22, 0.04) | 0.18 |
|  | 70-74 | -0.03 | (-0.38, 0.32) | 0.86 | 0.24 | (-0.09, 0.57) | 0.15 | -0.22 | (-0.55, 0.11) | 0.19 | -0.004 | (-0.26, 0.25) | 0.98 |
|  | 75-79 | -0.01 | (-0.39, 0.37) | 0.97 | 0.33 | (-0.18, 0.84) | 0.20 | -0.02 | (-0.40, 0.36) | 0.93 | 0.10 | (-0.21, 0.42) | 0.53 |
|  | ≥ 80 | -0.26 | (-0.64, 0.11) | 0.17 | -0.56 | (-0.95, -0.16) | 0.01 | -0.61 | (-0.98, -0.23) | <0.01 | -0.48 | (-0.79, -0.16) | <0.01 |
| Within 5 km | < 70 | 0.03 | (-0.19, 0.25) | 0.81 | -0.22 | (-0.43, -0.01) | 0.04 | 0.06 | (-0.16, 0.27) | 0.61 | -0.05 | (-0.21, 0.12) | 0.58 |
|  | 70-74 | 0.30 | (-0.24, 0.84) | 0.28 | -0.21 | (-0.73, 0.31) | 0.42 | 0.05 | (-0.46, 0.57) | 0.84 | 0.05 | (-0.35, 0.44) | 0.82 |
|  | 75-79 | -0.54 | (-1.05, -0.03) | 0.04 | -0.69 | (-1.37, -0.005) | 0.05 | -0.16 | (-0.68, 0.36) | 0.54 | -0.46 | (-0.88, -0.04) | 0.03 |
|  | ≥ 80 | 0.05 | (-0.60, 0.70) | 0.88 | 0.19 | (-0.51, 0.89) | 0.60 | 0.44 | (-0.23, 1.10) | 0.20 | 0.22 | (-0.34, 0.79) | 0.43 |

^1^Minimally adjusted for baseline age and sex. ^2^Fully adjusted for age, sex, education, census tract income, marital status, race/ethnicity, smoking status, alcohol consumption. Continuous distance interpreted per every 5 km closer a residence is to a lead facility. β: beta value for linear regression estimate, km: kilometers, STAR: Study of Healthy Aging in African Americans.

**Supplemental Table 13:** Sensitivity results for education stratified analysis adjusted linear regression associations between residential distance to lead releasing facility and baseline cognition in KHANDLE analytic sample (n=1,638). Cognitive measures are z-score standardized.

| **Distance to lead**  **facility** | **Education level** | **Cognitive measure** | | | | | | | | | | | |
| --- | --- | --- | --- | --- | --- | --- | --- | --- | --- | --- | --- | --- | --- |
|  |  | **Episodic memory** | | | **Semantic memory** | | | **Executive function** | | | **Global cognition** | | |
|  |  | **β** | **95% CI** | **P value** | **β** | **95% CI** | **P value** | **β** | **95% CI** | **P value** | **β** | **95% CI** | **P value** |
| **Minimally adjusted^1^** |  |  |  |  |  |  |  |  |  |  |  |  |  |
| Continuous (per 5 km) | ≤ high school | -0.10 | (-0.03, -0.17) | <0.01 | -0.17 | (-0.10, -0.24) | <0.01 | -0.07 | (-0.0002, -0.13) | 0.05 | -0.11 | (-0.06, -0.17) | <0.01 |
|  | trade school or college | -0.07 | (-0.03, -0.11) | <0.01 | -0.08 | (-0.04, -0.12) | <0.01 | -0.05 | (-0.01, -0.09) | 0.01 | -0.07 | (-0.04, -0.10) | <0.01 |
|  | graduate school | -0.06 | (-0.13, 0.01) | 0.10 | -0.02 | (-0.09, 0.05) | 0.67 | 0.05 | (-0.02, 0.12) | 0.19 | -0.01 | (-0.06, 0.05) | 0.76 |
| Within 1.5 km | ≤ high school | -0.11 | (-0.52, 0.30) | 0.58 | -0.32 | (-0.75, 0.12) | 0.15 | -0.20 | (-0.59, 0.20) | 0.33 | -0.21 | (-0.54, 0.12) | 0.22 |
|  | trade school or college | -0.17 | (-0.46, 0.11) | 0.24 | -0.17 | (-0.46, 0.13) | 0.26 | -0.35 | (-0.63, -0.06) | 0.02 | -0.23 | (-0.46, -0.002) | 0.05 |
|  | graduate school | 0.54 | (-0.30, 1.39) | 0.21 | -0.07 | (-0.94, 0.80) | 0.87 | -0.10 | (-0.99, 0.80) | 0.83 | 0.13 | (-0.55, 0.80) | 0.71 |
| Within 3 km | ≤ high school | -0.29 | (-0.52, -0.05) | 0.02 | -0.38 | (-0.62, -0.13) | <0.01 | -0.37 | (-0.60, -0.15) | <0.01 | -0.35 | (-0.53, -0.16) | <0.01 |
|  | trade school or college | -0.14 | (-0.28, -0.01) | 0.04 | -0.17 | (-0.30, -0.03) | 0.02 | -0.15 | (-0.28, -0.02) | 0.03 | -0.15 | (-0.26, -0.05) | <0.01 |
|  | graduate school | -0.15 | (-0.41, 0.11) | 0.26 | -0.36 | (-0.63, -0.10) | 0.01 | -0.06 | (-0.34, 0.21) | 0.65 | -0.19 | (-0.40, 0.01) | 0.07 |
| Within 5 km | ≤ high school | -0.37 | (-0.57, -0.17) | <0.01 | -0.55 | (-0.76, -0.34) | <0.01 | -0.34 | (-0.54, -0.15) | <0.01 | -0.42 | (-0.58, -0.26) | <0.01 |
|  | trade school or college | -0.24 | (-0.35, -0.13) | <0.01 | -0.24 | (-0.35, -0.13) | <0.01 | -0.15 | (-0.26, -0.04) | 0.01 | -0.21 | (-0.30, -0.12) | <0.01 |
|  | graduate school | -0.11 | (-0.29, 0.06) | 0.21 | -0.04 | (-0.22, 0.14) | 0.66 | 0.05 | (-0.13, 0.24) | 0.59 | -0.03 | (-0.17, 0.10) | 0.63 |
| **Fully adjusted^2^** |  |  |  |  |  |  |  |  |  |  |  |  |  |
| Continuous (per 5 km) | ≤ high school | -0.08 | (-0.01, -0.15) | 0.02 | -0.10 | (-0.04, -0.17) | <0.01 | -0.01 | (-0.08, 0.05) | 0.75 | -0.07 | (-0.01, -0.12) | 0.01 |
|  | trade school or college | -0.04 | (-0.004, -0.08) | 0.03 | -0.001 | (-0.04, 0.03) | 0.95 | 0.002 | (-0.03, 0.04) | 0.90 | -0.01 | (-0.04, 0.01) | 0.33 |
|  | graduate school | -0.04 | (-0.11, 0.02) | 0.20 | -0.01 | (-0.07, 0.05) | 0.65 | 0.04 | (-0.03, 0.10) | 0.24 | -0.01 | (-0.05, 0.04) | 0.79 |
| Within 1.5 km | ≤ high school | -0.01 | (-0.41, 0.39) | 0.95 | -0.33 | (-0.70, 0.03) | 0.08 | -0.14 | (-0.52, 0.23) | 0.45 | -0.16 | (-0.46, 0.14) | 0.29 |
|  | trade school or college | -0.13 | (-0.41, 0.15) | 0.37 | -0.05 | (-0.30, 0.21) | 0.72 | -0.23 | (-0.48, 0.03) | 0.08 | -0.13 | (-0.34, 0.07) | 0.19 |
|  | graduate school | 0.36 | (-0.46, 1.17) | 0.39 | -0.20 | (-0.91, 0.50) | 0.57 | -0.19 | (-0.97, 0.59) | 0.63 | -0.01 | (-0.57, 0.55) | 0.97 |
| Within 3 km | ≤ high school | -0.19 | (-0.44, 0.05) | 0.13 | -0.19 | (-0.41, 0.03) | 0.1 | -0.20 | (-0.43, 0.02) | 0.08 | -0.19 | (-0.38, -0.01) | 0.04 |
|  | trade school or college | -0.06 | (-0.20, 0.07) | 0.37 | -0.0007 | (-0.12, 0.12) | 0.99 | -0.01 | (-0.14, 0.11) | 0.82 | -0.03 | (-0.12, 0.07) | 0.60 |
|  | graduate school | -0.09 | (-0.35, 0.17) | 0.48 | -0.27 | (-0.50, -0.05) | 0.02 | 0.03 | (-0.22, 0.27) | 0.84 | -0.11 | (-0.29, 0.06) | 0.21 |
| Within 5 km | ≤ high school | -0.29 | (-0.51, -0.07) | 0.01 | -0.36 | (-0.56, -0.15) | <0.01 | -0.14 | (-0.35, 0.07) | 0.18 | -0.26 | (-0.43, -0.10) | <0.01 |
|  | trade school or college | -0.16 | (-0.27, -0.05) | <0.01 | -0.03 | (-0.13, 0.07) | 0.56 | 0.002 | (-0.10, 0.11) | 0.97 | -0.06 | (-0.14, 0.02) | 0.12 |
|  | graduate school | -0.06 | (-0.24, 0.12) | 0.51 | -0.01 | (-0.16, 0.15) | 0.93 | 0.06 | (-0.11, 0.22) | 0.52 | -0.004 | (-0.12, 0.12) | 0.95 |

^1^Minimally adjusted for baseline age and sex. ^2^Fully adjusted for age, sex, education, census tract income, marital status, race/ethnicity, smoking status, alcohol consumption. Continuous distance interpreted per every 5 km closer a residence is to a lead facility. β: beta value for linear regression estimate, km: kilometers, KHANDLE: Kaiser Healthy Aging and Diverse Life Experiences Study.

**Supplemental Table 14:** Sensitivity results for education stratified analysis adjusted linear regression associations between residential distance to lead releasing facility and baseline cognition in STAR analytic sample (n=741). Cognitive measures are z-score standardized.

| **Distance to lead**  **facility** | **Education level** | **Cognitive measure** | | | | | | | | | | | |
| --- | --- | --- | --- | --- | --- | --- | --- | --- | --- | --- | --- | --- | --- |
|  |  | **Episodic memory** | | | **Semantic memory** | | | **Executive function** | | | **Global cognition** | | |
|  |  | **β** | **95% CI** | **P value** | **β** | **95% CI** | **P value** | **β** | **95% CI** | **P value** | **β** | **95% CI** | **P value** |
| **Minimally adjusted** ^1^ |  |  |  |  |  |  |  |  |  |  |  |  |  |
| Continuous (per 5 km) | ≤ high school | -0.14 | (-0.61, 0.32) | 0.55 | -0.18 | (-0.70, 0.34) | 0.50 | -0.23 | (-0.69, 0.23) | 0.33 | -0.18 | (-0.55, 0.18) | 0.33 |
|  | trade school or college | -0.09 | (-0.27, 0.09) | 0.35 | -0.15 | (-0.33, 0.04) | 0.12 | -0.05 | (-0.22, 0.12) | 0.56 | -0.09 | (-0.23, 0.04) | 0.18 |
|  | graduate school | 0.002 | (-0.14, 0.15) | 0.97 | 0.03 | (-0.12, 0.17) | 0.73 | 0.02 | (-0.11, 0.16) | 0.76 | 0.02 | (-0.09, 0.12) | 0.77 |
| Within 1.5 km | ≤ high school | -0.18 | (-0.53, 0.17) | 0.32 | -0.21 | (-0.59, 0.18) | 0.30 | -0.19 | (-0.53, 0.16) | 0.28 | -0.19 | (-0.47, 0.08) | 0.17 |
|  | trade school or college | -0.08 | (-0.32, 0.16) | 0.53 | 0.06 | (-0.19, 0.30) | 0.64 | 0.04 | (-0.19, 0.27) | 0.74 | 0.01 | (-0.18, 0.19) | 0.95 |
|  | graduate school | -0.27 | (-1.10, 0.56) | 0.53 | -0.35 | (-1.17, 0.48) | 0.41 | 0.25 | (-0.51, 1.01) | 0.52 | -0.12 | (-0.73, 0.49) | 0.70 |
| Within 3 km | ≤ high school | -0.07 | (-0.35, 0.21) | 0.62 | -0.20 | (-0.5, 0.11) | 0.21 | -0.28 | (-0.55, -0.01) | 0.04 | -0.18 | (-0.40, 0.04) | 0.10 |
|  | trade school or college | -0.07 | (-0.22, 0.09) | 0.40 | -0.06 | (-0.22, 0.10) | 0.45 | -0.08 | (-0.22, 0.07) | 0.31 | -0.07 | (-0.18, 0.05) | 0.26 |
|  | graduate school | -0.07 | (-0.37, 0.24) | 0.68 | -0.005 | (-0.31, 0.30) | 0.98 | 0.01 | (-0.27, 0.29) | 0.94 | -0.02 | (-0.25, 0.21) | 0.86 |
| Within 5 km | ≤ high school | -0.04 | (-0.53, 0.45) | 0.87 | -0.10 | (-0.64, 0.44) | 0.71 | -0.19 | (-0.67, 0.28) | 0.42 | -0.11 | (-0.50, 0.27) | 0.57 |
|  | trade school or college | -0.0050 | (-0.21, 0.20) | 0.97 | -0.24 | (-0.45, -0.03) | 0.03 | 0.01 | (-0.19, 0.21) | 0.95 | -0.08 | (-0.24, 0.08) | 0.34 |
|  | graduate school | -0.11 | (-0.46, 0.23) | 0.52 | -0.10 | (-0.44, 0.24) | 0.57 | 0.05 | (-0.27, 0.36) | 0.76 | -0.05 | (-0.31, 0.20) | 0.67 |
| **Fully adjusted^2^** |  |  |  |  |  |  |  |  |  |  |  |  |  |
| Continuous (per 5 km) | ≤ high school | -0.03 | (0.48, -0.54) | 0.90 | -0.33 | (-0.87, 0.21) | 0.23 | -0.28 | (-0.76, 0.20) | 0.25 | -0.21 | (0.17, -0.60) | 0.28 |
|  | trade school or college | -0.04 | (0.16, -0.23) | 0.72 | -0.14 | (-0.34, 0.06) | 0.16 | 0.04 | (-0.15, 0.22) | 0.70 | -0.05 | (0.10, -0.20) | 0.53 |
|  | graduate school | 0.002 | (-0.15, 0.15) | 0.98 | 0.02 | (-0.12, 0.17) | 0.74 | 0.02 | (-0.12, 0.15) | 0.82 | 0.01 | (0.12, -0.09) | 0.80 |
| Within 1.5 km | ≤ high school | -0.84 | (-2.60, 0.92) | 0.35 | -1.69 | (-3.55, 0.17) | 0.07 | -1.07 | (-2.73, 0.59) | 0.21 | -1.20 | (-2.52, 0.13) | 0.08 |
|  | trade school or college | -0.06 | (-0.31, 0.18) | 0.62 | 0.08 | (-0.17, 0.33) | 0.52 | 0.07 | (-0.16, 0.30) | 0.54 | 0.03 | (-0.16, 0.21) | 0.75 |
|  | graduate school | -0.26 | (-1.10, 0.58) | 0.55 | -0.38 | (-1.21, 0.45) | 0.37 | 0.24 | (-0.54, 1.01) | 0.55 | -0.13 | (-0.75, 0.48) | 0.67 |
| Within 3 km | ≤ high school | 0.02 | (-0.28, 0.31) | 0.92 | -0.24 | (-0.55, 0.07) | 0.13 | -0.29 | (-0.57, -0.02) | 0.03 | -0.17 | (-0.39, 0.05) | 0.13 |
|  | trade school or college | -0.04 | (-0.21, 0.12) | 0.60 | -0.06 | (-0.23, 0.11) | 0.48 | -0.03 | (-0.19, 0.13) | 0.70 | -0.05 | (-0.17, 0.08) | 0.48 |
|  | graduate school | -0.14 | (-0.48, 0.20) | 0.42 | 0.02 | (-0.32, 0.35) | 0.92 | 0.04 | (-0.27, 0.36) | 0.78 | -0.03 | (-0.27, 0.22) | 0.84 |
| Within 5 km | ≤ high school | 0.12 | (-0.46, 0.69) | 0.69 | -0.15 | (-0.76, 0.46) | 0.63 | -0.2 | (-0.74, 0.35) | 0.48 | -0.08 | (-0.51, 0.36) | 0.73 |
|  | trade school or college | 0.06 | (-0.17, 0.28) | 0.61 | -0.25 | (-0.48, -0.03) | 0.03 | 0.11 | (-0.10, 0.32) | 0.32 | -0.03 | (-0.20, 0.14) | 0.74 |
|  | graduate school | -0.18 | (-0.56, 0.21) | 0.37 | -0.06 | (-0.44, 0.32) | 0.76 | 0.11 | (-0.24, 0.46) | 0.55 | -0.04 | (-0.32, 0.24) | 0.76 |

^1^Minimally adjusted for baseline age and sex. ^2^Fully adjusted for age, sex, education, census tract income, marital status, race/ethnicity, smoking status, alcohol consumption. Continuous distance interpreted per every 5 km closer a residence is to a lead facility. β: beta value for linear regression estimate, km: kilometers, STAR: Study of Healthy Aging in African Americans.

**Supplemental Table 15**. Sensitivity results restricted to street level residential address adjusted linear regression associations between residential distance to lead releasing facility and baseline cognition in KHANDLE analytic sample (n=1,477). Cognitive measures are z-score standardized.

| **Distance to lead**  **facility** | **Cognitive measure** | | | | | | | | | | | |
| --- | --- | --- | --- | --- | --- | --- | --- | --- | --- | --- | --- | --- |
|  | **Episodic memory** | | | **Semantic memory** | | | **Executive function** | | | **Global cognition** | | |
|  | **β** | **95% CI** | **P value** | **β** | **95% CI** | **P value** | **β** | **95% CI** | **P value** | **β** | **95% CI** | **P value** |
| **Minimally adjusted^1^** |  |  |  |  |  |  |  |  |  |  |  |  |
| Continuous (per 5 km) | -0.09 | (-0.06, -0.12) | <0.01 | -0.08 | (-0.05, -0.12) | <0.01 | -0.04 | (-0.002, -0.07) | 0.04 | -0.07 | (-0.04, -0.10) | <0.01 |
| Within 1.5 km | -0.25 | (-0.49, -0.02) | 0.03 | -0.41 | (-0.65, -0.17) | <0.01 | -0.48 | (-0.73, -0.24) | <0.01 | -0.38 | (-0.57, -0.19) | <0.01 |
| Within 3 km | -0.24 | (-0.35, -0.12) | <0.01 | -0.32 | (-0.43, -0.20) | <0.01 | -0.26 | (-0.38, -0.14) | <0.01 | -0.27 | (-0.36, -0.18) | <0.01 |
| Within 5 km | -0.25 | (-0.34, -0.16) | <0.01 | -0.26 | (-0.35, -0.16) | <0.01 | -0.16 | (-0.25, -0.06) | <0.01 | -0.22 | (-0.30, -0.15) | <0.01 |
| **Fully adjusted^2^** |  |  |  |  |  |  |  |  |  |  |  |  |
| Continuous (per 5 km) | -0.06 | (-0.01, -0.09) | <0.01 | -0.02 | (-0.05, 0.01) | 0.12 | 0.01 | (-0.02, 0.04) | 0.52 | -0.02 | (-0.05, -0.001) | 0.04 |
| Within 1.5 km | -0.11 | (-0.34, 0.12) | 0.35 | -0.20 | (-0.40, 0.01) | 0.06 | -0.26 | (-0.47, -0.05) | 0.02 | -0.19 | (-0.35, 0.02) | 0.02 |
| Within 3 km | -0.10 | (-0.21, 0.01) | 0.08 | -0.10 | (-0.20, 0.01) | 0.07 | -0.04 | (-0.15, 0.07) | 0.47 | -0.08 | (-0.16, 0.004) | 0.06 |
| Within 5 km | -0.15 | (-0.24, -0.06) | <0.01 | -0.06 | (-0.14, 0.02) | 0.16 | 0.01 | (-0.08, 0.09) | 0.88 | -0.07 | (-0.13, -0.002) | 0.04 |

^1^Minimally adjusted for age and sex. ^2^Fully adjusted for age, sex, education, census tract income, marital status, race/ethnicity, smoking status, alcohol consumption. Complete case: non-missing values for all observations required for study inclusion. Continuous distance interpreted per every 5 km closer a residence is to a lead facility. β: beta value for linear regression estimate, km: kilometers, KHANDLE: Kaiser Healthy Aging and Diverse Life Experiences Study.

**Supplemental Table 16**. Sensitivity results restricted to street level residential address analysis adjusted linear regression associations between residential distance to lead releasing facility and baseline cognition in STAR analytic sample (n=687). Cognitive measures are z-score standardized.

| **Distance to lead**  **facility** | **Cognitive measure** | | | | | | | | | | | |
| --- | --- | --- | --- | --- | --- | --- | --- | --- | --- | --- | --- | --- |
|  | **Episodic memory** | | | **Semantic memory** | | | **Executive function** | | | **Global cognition** | | |
|  | **β** | **95% CI** | **P value** | **β** | **95% CI** | **P value** | **β** | **95% CI** | **P value** | **β** | **95% CI** | **P value** |
| **Minimally adjusted^1^** |  |  |  |  |  |  |  |  |  |  |  |  |
| Continuous (per 5 km) | -0.08 | (-0.19, 0.04) | 0.17 | -0.12 | (-0.003, -0.24) | 0.04 | -0.10 | (-0.22, 0.04) | 0.07 | -0.10 | (-0.01, -0.19) | 0.03 |
| Within 1.5 km | -0.21 | (-0.41, -0.01) | 0.04 | -0.20 | (-0.41, 0.01) | 0.06 | -0.19 | (-0.38, 0.01) | 0.06 | -0.20 | (-0.36, -0.04) | 0.01 |
| Within 3 km | -0.10 | (-0.23, 0.03) | 0.15 | -0.13 | (-0.27, -0.01) | 0.06 | -0.16 | (-0.28, -0.03) | 0.02 | -0.13 | (-0.23, -0.02) | 0.02 |
| Within 5 km | -0.09 | (-0.27, 0.08) | 0.30 | -0.30 | (-0.48, -0.12) | <0.01 | -0.10 | (-0.27, 0.087 | 0.27 | -0.16 | (-0.30, -0.03) | 0.02 |
| **Fully adjusted^2^** |  |  |  |  |  |  |  |  |  |  |  |  |
| Continuous (per 5 km) | -0.03 | (-0.14, 0.09) | 0.62 | -0.07 | (-0.19, 0.04) | 0.23 | -0.03 | (-0.02, -0.14) | 0.58 | -0.04 | (-0.13, 0.04) | 0.32 |
| Within 1.5 km | -0.13 | (-0.33, 0.07) | 0.07 | -0.10 | (-0.31, 0.11) | 0.34 | -0.07 | (-0.26, 0.12) | 0.48 | -0.10 | (-0.25, 0.05) | 0.19 |
| Within 3 km | -0.04 | (-0.18, 0.10) | 0.55 | -0.09 | (-0.24, 0.05) | 0.21 | -0.07 | (-0.20, 0.037 | 0.32 | -0.07 | (-0.17, 0.04) | 0.21 |
| Within 5 km | 0.02 | (-0.17, 0.21) | 0.84 | -0.24 | (-0.43, -0.05) | 0.02 | 0.08 | (-0.10, 0.26) | 0.39 | -0.05 | (-0.19, 0.10) | 0.52 |

^1^Minimally adjusted for age and sex. ^2^Fully adjusted for age, sex, education, census tract income, marital status, race/ethnicity, smoking status, alcohol consumption. Complete case: non-missing values for all observations required for study inclusion. Continuous distance interpreted per every 5 km closer a residence is to a lead facility. β: beta value for linear regression estimate, km: kilometers, STAR: Study of Healthy Aging in African Americans.

**Supplemental Figure 3.** Forest plot of mean difference 95% confidence intervals for minimally adjusted models in the KHANDLE and STAR analytic samples, and meta-analyzed across cohorts.


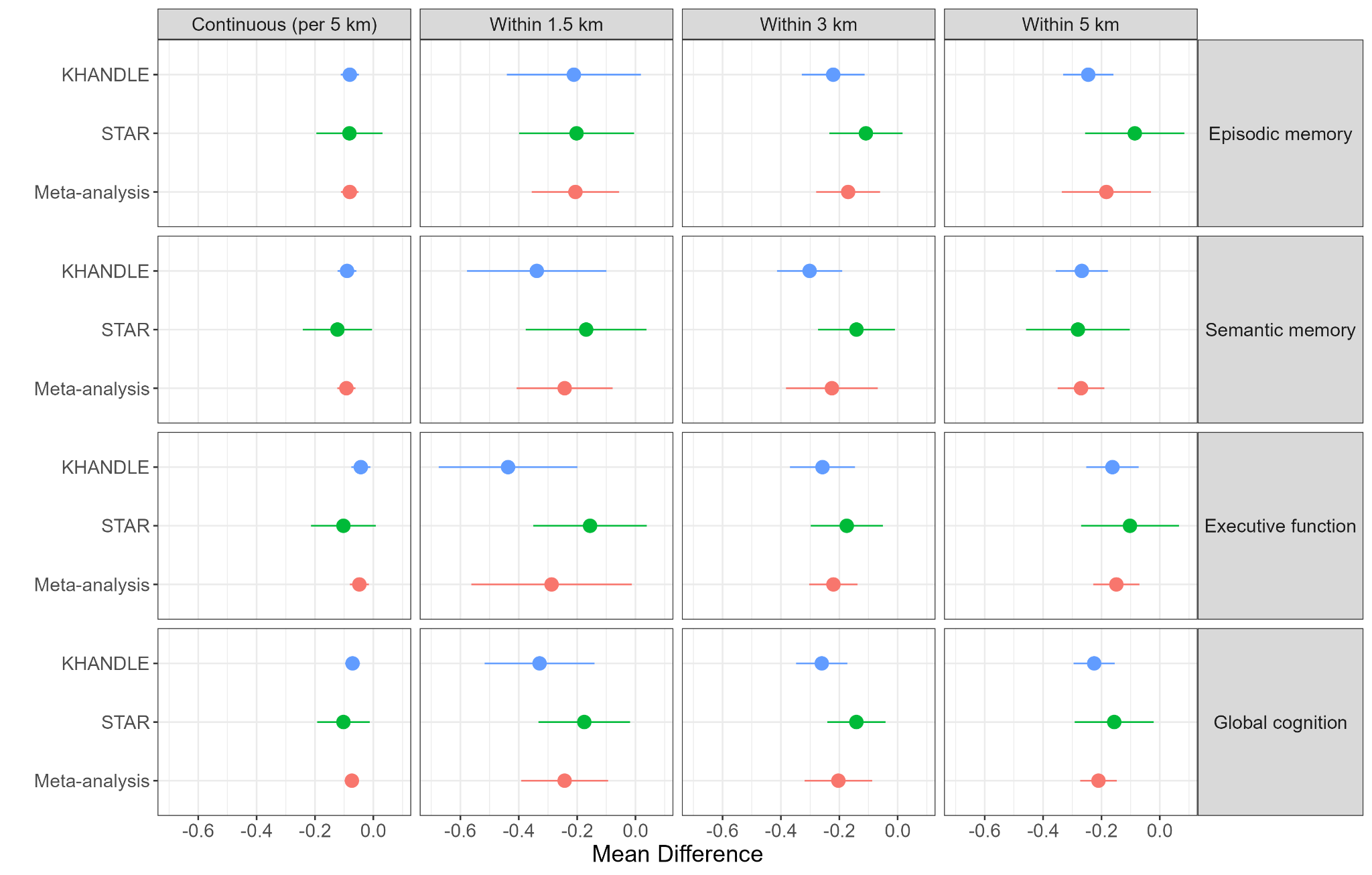


Minimally adjusted for age and sex. KHANDLE: Kaiser Healthy Aging and Diverse Life Experiences Study; STAR: Study of Healthy Aging in African Americans

**Supplemental Table 17.** Kaiser Healthy Aging and Diverse Life Experiences Study (KHANDLE) analytic sample descriptive statistics by residential distance buffer around a lead releasing facility releasing ≥ 100lbs lead.

|  |  | **Residential distance buffer** | | | | | | | | |
| --- | --- | --- | --- | --- | --- | --- | --- | --- | --- | --- |
| **Characteristic** | **Overall**  N =1,638 | **Lead release >=100 within 1.5km** | | | **Lead release >=100 within 3km** | | | **Lead release >=100 within 5km** | | |
|  |  | **No**  N = 1,598 | **Yes**  N = 40 | ***p* value^1^** | **No**  N = 1,393 | **Yes**  N = 254 | ***p* value^1^** | **No**  N = 1,137 | **Yes**  N = 501 | ***p* value*^1^*** |
| **Global cognition^2^** | 0.01 (0.81) | 0.02 (0.81) | -0.32 (0.94) | 0.03 | 0.06 (0.80) | -0.24 (0.86) | <0.01 | 0.05 (0.79) | -0.08 (0.87) | <0.01 |
| **Executive functioning^2^** | 0.01 (1.0) | 0.02 (0.99) | -0.48 (1.11) | <0.01 | 0.06 (0.98) | -0.23 (1.04) | <0.01 | 0.03 (0.98) | -0.03 (1.04) | 0.23 |
| **Episodic memory^2^** | 0.01 (1.0) | 0.01 (0.99) | -0.10 (1.16) | 0.55 | 0.04 (0.99) | -0.18 (1.02) | <0.01 | 0.05 (0.99) | -0.08 (1.02) | 0.01 |
| **Semantic memory^2^** | 0.02 (0.99) | 0.03 (0.99) | -0.38 (1.03) | 0.01 | 0.07 (0.98) | -0.30 (1.04) | <0.01 | 0.08 (0.97) | -0.12 (1.04) | <0.01 |
| **Average distance to facility (km)** | 8.2 (6.9) | 8.4 (6.9) | 1.1 (0.3) | <0.01 | 9.3 (6.9) | 2.1 (0.6) | <0.01 | 10.5 (7.2) | 3.1 (1.1) | <0.01 |
| **Total lead releases (lbs)^3^** | 2,313.6 (5,344.3) | 2,299.9 (5,353.9) | 2,859.1 (4,978.3) | 0.48 | 1,918.3 (4,532.1) | 4,561.4  (8,275.2) | <0.01 | 1,300.4 (3,762.3) | 4,613.0 (7,329.3) | <0.01 |
| **Air lead releases (lbs)** | 11.1 (29.8) | 10.8 (28.9) | 25.4 (51.4) | 0.08 | 8.8 (25.5) | 24.3 (45.0) | <0.01 | 4.8 (15.8) | 25.4 (45.2) | <0.01 |
| **Water lead releases (lbs)** | 1.6 (7.2) | 1.5 (7.2) | 1.9 (9.1) | 0.81 | 1.5 (7.2) | 1.6 (7.6) | 0.88 | 1.5 (7.1) | 1.7 (7.6) | 0.54 |
| **Land Lead releases (lbs)** | 10.4 (232.5) | 10.48 (235.3) | 5.9 (37.5) | 0.58 | 11.4 (251.8) | 4.5 (31.4) | 0.32 | 13.5 (278.5) | 3.2 (26.6) | 0.40 |
| **Age at interview** | 76.1 (7.1) | 76.1 (7.1) | 75.4 (6.2) | 0.47 | 76.0 (7.1) | 76.2 (7.4) | 0.71 | 76.2 (7.1) | 75.8 (7.1) | 0.25 |
| **Sex** |  |  |  | 0.88 |  |  | 0.27 |  |  | 0.03 |
| Male | 674 (41.1%) | 658 (41.2%) | 16 (40.0%) |  | 581 (41.7%) | 93 (38.0%) |  | 488 (42.9%) | 186 (37.1%) |  |
| Female | 964 (58.9%) | 940 (58.8%) | 24 (60.0%) |  | 812 (58.3%) | 152 (62.0%) |  | 649 (57.1%) | 315 (62.9%) |  |
| **Race/ethnicity** |  |  |  | 0.60 |  |  | <0.01 |  |  | <0.01 |
| Asian | 410 (25.0%) | 398 (24.9%) | 12 (30.0%) |  | 357 (25.6%) | 53 (21.6%) |  | 295 (25.9%) | 115 (23.0%) |  |
| Black | 422 (25.8%) | 413 (25.8%) | 9 (22.5%) |  | 333 (23.9%) | 89 (36.3%) |  | 246 (21.6%) | 176 (35.1%) |  |
| LatinX | 322 (19.7%) | 312 (19.5%) | 10 (25.0%) |  | 276 (19.2%) | 55 (22.4%) |  | 225 (19.8%) | 97 (19.4%) |  |
| White | 484 (29.5%) | 475 (29.7%) | 9 (22.5%) |  | 436 (31.3%) | 48 (19.6%) |  | 371 (32.6%) | 113 (22.6%) |  |
| **Education** |  |  |  | 0.02 |  |  | <0.01 |  |  | 0.07 |
| ≤ High School | 274 (16.7%) | 262 (16.4%) | 12 (30.0%) |  | 217 (15.4%) | 57 (23.3%) |  | 178 (15.7%) | 96 (19.2%) |  |
| > High School | 1364 (83.3%) | 1336 (83.6%) | 28 (70.0%) |  | 1,176 (84.4%) | 188 (76.7%) |  | 959 (84.3%) | 405 (80.8%) |  |
| **Marital Status** |  |  |  | 0.22 |  |  | <0.01 |  |  | <0.01 |
| Married/ living as married | 933 (57.0%) | 914 (57.2%) | 19 (47.5%) |  | 825 (59.2%) | 108 (44.1%) |  | 672 (59.1%) | 261 (52.1%) |  |
| Not married | 705 (43.0%) | 684 (42.8%) | 21 (52.5%) |  | 568 (40.8%) | 137 (55.9%) |  | 465 (40.9%) | 240 (47.9%) |  |
| **Average census tract income** | 121,338 (52,304) | 122,237 (52,696) | 98,476 (28,950) | <0.01 | 126,415 (54,090) | 92,466 (26,045) | <0.01 | 126,482 (53,645) | 109,662 (47,132) | <0.01 |
| **Smoking status** |  |  |  | 0.97 |  |  | 0.39 |  |  | 0.16 |
| Never | 912 (55.7%) | 890 (55.7%) | 22 (55.0%) |  | 774 (55.6%) | 138 (56.3%) |  | 632 (55.6%) | 280 (55.9%) |  |
| Past | 677 (41.3%) | 660 (41.3%) | 17 (42.5%) |  | 574 (41.2%) | 103 (42.0%) |  | 465 (40.9%) | 212 (42.3%) |  |
| Current | 49 (3.0%) | 48 (3.0%) | 1 (2.5%) |  | 45 (3.2%) | 4 (1.6%) |  | 40 (3.5%) | 9 (1.8%) |  |
| **Alcohol consumption** |  |  |  | 0.08 |  |  | 0.07 |  |  | 0.08 |
| Never | 481 (29.4%) | 465 (29.1%) | 16 (40.0%) |  | 400 (28.7%) | 81 (33.1%) |  | 318 (28.0%) | 163 (32.5%) |  |
| Less than once a week | 572 (34.9%) | 558 (34.9%) | 14 (35.0%) |  | 478 (34.3%) | 94 (38.4%) |  | 393 (34.6%) | 179 (35.7%) |  |
| 1 - 6 days per week | 425 (25.9%) | 421 (26.3%) | 4 (10.0%) |  | 377 (27.1%) | 48 (19.6%) |  | 314 (27.6%) | 111 (22.2%) |  |
| Every day | 160 (9.8%) | 154 (9.6%) | 6 (15.0%) |  | 138 (9.9%) | 22 (9.0%) |  | 112 (9.9%) | 48 (9.6%) |  |

Mean (SD) for continuous variables, N(%) categorical. ^1^Fishers exact test, Satterthwaite t-test. ^2^Cognitive measures are z-score standardized. ^3^Total lead releases is a combination of air, land, water, and off-site lead releases reported by the facility to the Toxics Release Inventory. Off-site releases was not included in this analysis and therefore when combined, proportions of lead released through air, water, and land may not add to the total lead release number listed. Average census tract income rounded to the nearest whole number. lbs: pounds, km: kilometer

**Supplemental Table 18.** Study of Healthy Aging in African Americans (STAR) analytic sample descriptive statistics by residential distance buffer around a lead releasing facility releasing ≥ 100lbs lead.

|  |  | **Residential distance buffer** | | | | | | | | |
| --- | --- | --- | --- | --- | --- | --- | --- | --- | --- | --- |
| **Characteristic** | **Overall**  N = 741 | **Lead release >=100 within 1.5 km** | | | **Lead release >=100 within 3 km** | | | **Lead release >=100 within 5 km** | | |
|  |  | **No**  N = 670 | **Yes**  N = 71 | ***p* value*^1^*** | **No**  N = 438 | **Yes**  N = 303 | ***p* value*^1^*** | **No**  N = 207 | **Yes**  N = 534 | ***p* value*^1^*** |
| **Global cognition** | 0.01 (0.81) | 0.04 (0.80) | -0.23 (0.87) | 0.02 | 0.08 (0.79) | -0.09 (0.82) | <0.01 | 0.11 (0.78) | -0.03 (0.82) | 0.03 |
| **Executive functioning** | 0.01 (0.99) | 0.04 (0.98) | -0.22 (1.08) | 0.06 | 0.10 (0.97) | -0.10 (1.01) | <0.01 | 0.09 (0.97) | -0.02 (1.00) | 0.16 |
| **Episodic memory** | 0.02 (0.99) | 0.05 (0.99) | -0.25 (0.97) | 0.01 | 0.07 (1.0) | -0.06 (0.99) | 0.06 | 0.10 (0.95) | -0.01 (1.01) | 0.16 |
| **Semantic memory** | 0.00 (0.99) | 0.02 (0.97) | -0.21 (1.13) | 0.09 | 0.07 (0.97) | -0.09 (1.02) | 0.03 | 0.13 (1.02) | -0.05 (0.97) | 0.02 |
| **Average distance to facility (km)** | 3.6 (2.8) | 3.8 (2.8) | 1.1 (0.3) | <0.01 | 4.6 (3.2) | 2.1 (0.7) | <0.01 | 5.5 (4.4) | 2.8 (1.1) | <0.01 |
| **Total lead releases (lbs)^3^** | 4,971.3 (6,251.5) | 5,035.5 (6,283.5) | 4,364.9 (5,948.5) | 0.37 | 4,544.5 (6,142.5) | 5,588.3 (6,365.7) | 0.02 | 2,618.9 (5,202.2) | 5,883.1 (6,388.8) | <0.01 |
| **Air lead releases (lbs)** | 27.1 (36.9) | 27.8 (37.2) | 25.1 (36.5) | 0.63 | 24.7 (36.0) | 30.5 (38.1) | 0.03 | 14.5 (29.8) | 31.9 (38.4) | <0.01 |
| **Water lead releases (lbs)** | 0.8 (3.3) | 0.8 (3.4) | 0.4 (1.1) | 0.04 | 1.1 (4.1) | 0.4 (1.0) | <0.01 | 1.5 (5.6) | 0.5 (1.6) | <0.01 |
| **Land lead releases (lbs)** | 0.2 (4.6) | 0.2 (4.9) | 0.0 (0.0) | 0.28 | 0.3 (6.0) | 0.0 (0.0) | 0.28 | 0.0 (0.5) | 0.2 (5.5) | 0.43 |
| **Age at interview** | 68.8 (8.8) | 68.6 (8.7) | 69.9 (9.8) | 0.29 | 68.2 (8.7) | 69.4 (9.1) | 0.07 | 67.1 (8.3) | 69.4 (9.0) | <0.01 |
| **Sex** |  |  |  | 0.72 |  |  | 0.31 |  |  | 0.29 |
| Male | 233 (31.4%) | 212 (31.6%) | 21 (29.6%) |  | 144 (32.9%) | 89 (29.4%) |  | 71 (34.4%) | 162 (30.3%) |  |
| Female | 508 (68.6%) | 458 (68.4%) | 50 (70.4%) |  | 294 (67.1%) | 214 (70.6%) |  | 136 (65.7%) | 372 (69.7%) |  |
| **Education** |  |  |  | 0.02 |  |  | 0.35 |  |  | 0.49 |
| ≤ High School | 135 (18.2%) | 115 (17.2%) | 20 (28.2%) |  | 75 (17.1%) | 60 (19.8%) |  | 41 (19.8%) | 94 (17.6%) |  |
| > High School | 606 (81.8%) | 555 (82.8%) | 51 (71.8%) |  | 363 (82.9%) | 243 (80.2%) |  | 166 (80.2%) | 440 (82.4%) |  |
| **Marital Status** |  |  |  | <0.01 |  |  | 0.13 |  |  | 0.58 |
| Married/ living as married | 328 (44.3%) | 307 (45.8%) | 21 (29.6%) |  | 204 (46.6%) | 124 (40.9%) |  | 95 (45.9%) | 233 (43.6%) |  |
| Not married | 413 (55.7%) | 363 (54.2%) | 50 (70.4%) |  | 234 (53.4%) | 179 (59.1%) |  | 112 (54.1%) | 301 (56.4%) |  |
| **Average census tract income** | 103,014 (48,533) | 105,240 (49,963) | 82,003 (2,300) | <0.01 | 117,367 (56,269.9) | 82,266 (21,433) | <0.01 | 125,247 (64,910) | 94,396 (37,087) | <0.01 |
| **Smoking status** |  |  |  | 0.66 |  |  | 0.12 |  |  | 0.42 |
| Never | 391 (52.8%) | 357 (53.3%) | 34 (47.9%) |  | 244 (55.7%) | 147 (48.5%) |  | 117 (56.5%) | 274 (51.3%) |  |
| Past | 307 (41.4%) | 275 (41.8%) | 32 (45.1%) |  | 168 (38.4%) | 139 (45.9%) |  | 78 (37.7%) | 229 (42.9%) |  |
| Current | 43 (5.8%) | 38 (5.7%) | 5 (7.0%) |  | 26 (5.9%) | 17 (5.6%) |  | 12 (5.8%) | 31 (5.8%) |  |
| **Alcohol consumption** |  |  |  | 0.33 |  |  | 0.02 |  |  | 0.09 |
| Never | 270 (36.4%) | 241 (36.0%) | 29 (40.8%) |  | 165 (37.7%) | 105 (34.7%) |  | 76 (36.7%) | 194 (36.3%) |  |
| Less than once a week | 237 (32.0%) | 216 (32.2%) | 21 (29.6%) |  | 124 (28.3%) | 113 (37.3%) |  | 54 (26.1%) | 183 (34.3%) |  |
| 1 - 6 days per week | 208 (28.1%) | 187 (27.9%) | 21 (29.6%) |  | 129 (29.5%) | 79 (26.1%) |  | 67 (32.4%) | 141 (26.4%) |  |
| Every day | 26 (3.5%) | 26 (3.9%) | 0.0 (0.0%) |  | 20 (4.6%) | 6 (2.0%) |  | 10 (4.8%) | 16 (3.0%) |  |

Mean (SD) for continuous variables, N(%) categorical. ^1^Fishers exact test, Satterthwaite t-test. ^2^Cognitive measures are z-score standardized.^3^Total lead releases is a combination of air, land, water, and off-site lead releases reported by the facility to the Toxics Release Inventory. Off-site releases was not included in this analysis and therefore when combined, proportions of lead released through air, water, and land may not add to the total lead release number listed. Average census tract income rounded to the nearest whole number. lbs: pounds, km: kilometer

**Supplemental Table 19**. Sensitivity results for analysis restricted to facilities releasing >= 100 lbs of lead adjusted linear regression associations between residential distance to lead releasing facility and baseline cognition in KHANDLE analytic sample. Cognitive measures are z-score standardized.

| **Distance to lead**  **facility** | **Cognitive measure** | | | | | | | | | | | |
| --- | --- | --- | --- | --- | --- | --- | --- | --- | --- | --- | --- | --- |
|  | **Episodic memory** | | | **Semantic memory** | | | **Executive function** | | | **Global cognition** | | |
|  | **β** | **95% CI** | ***p***  **value** | **β** | **95% CI** | ***p* value** | **β** | **95% CI** | ***p* value** | **β** | **95% CI** | ***p* value** |
| Continuous (per 5 km)^1^ | -0.11 | (-0.16, -0.06) | <0.01 | -0.04 | (-0.09, -0.001) | 0.05 | 0.02 | (-0.03, 0.06) | 0.52 | -0.04 | (-0.08, -0.01) | 0.02 |
| Within 1.5 km^2^ | -0.03 | (-0.30, 0.23) | 0.80 | -0.24 | (-0.48, -0.001) | 0.05 | -0.34 | (-0.59, -0.09) | 0.01 | -0.21 | (-0.40, -0.01) | 0.04 |
| Within 3 km^2^ | -0.12 | (-0.24, 0.003) | 0.06 | -0.13 | (-0.23, -0.02) | 0.02 | -0.07 | (-0.18, 0.04) | 0.22 | -0.10 | (-0.19, -0.02) | 0.02 |
| Within 5 km^2^ | -0.11 | (-0.20, -0.20) | 0.02 | -0.02 | (-0.11, 0.06) | 0.56 | 0.04 | (-0.05, 0.12) | 0.38 | -0.03 | (-0.10, 0.03) | 0.33 |

All models fully adjusted for age, sex, education, census tract income, marital status, race/ethnicity, smoking status, and alcohol consumption. β: beta value for linear regression estimate, km: kilometers.

^1^Sample size n = 979

^2^Sample size n = 1,638

**Supplemental Table 20**. Sensitivity results for analysis restricted to facilities releasing >= 100 lbs of lead adjusted linear regression associations between residential distance to lead releasing facility and baseline cognition in STAR analytic sample. Cognitive measures are z-score standardized.

| **Distance to lead**  **facility** | **Cognitive measure** | | | | | | | | | | | |
| --- | --- | --- | --- | --- | --- | --- | --- | --- | --- | --- | --- | --- |
|  | **Episodic memory** | | | **Semantic memory** | | | **Executive function** | | | **Global cognition** | | |
|  | **β** | **95% CI** | ***p***  **value** | **β** | **95% CI** | ***p* value** | **β** | **95% CI** | ***p* value** | **β** | **95% CI** | ***p* value** |
| Continuous (per 5 km)^1^ | -0.01 | (-0.21, 0.19) | 0.91 | -0.07 | (-0.27, 0.14) | 0.54 | -0.04 | (-0.24, 0.15) | 0.67 | -0.04 | (-0.20, 0.12) | 0.62 |
| Within 1.5 km^2^ | -0.18 | (-0.39, 0.03) | 0.09 | -0.10 | (-0.32, 0.11) | 0.35 | -0.09 | (-0.29, 0.11) | 0.38 | -0.13 | (-0.29, 0.04) | 0.13 |
| Within 3 km^2^ | -0.07 | (-0.20, 0.07) | 0.31 | -0.09 | (-0.23, 0.05) | 0.21 | -0.09 | (-0.22, 0.04) | 0.19 | -0.08 | (-0.19, 0.02) | 0.12 |
| Within 5 km^2^ | -0.01 | (-0.16, 0.13) | 0.87 | -0.10 | (-0.24, 0.05) | 0.21 | 0.05 | (-0.09, 0.19) | 0.47 | -0.02 | (-0.13, 0.09) | 0.74 |

All models fully adjusted for age, sex, education, census tract income, marital status, race/ethnicity, smoking status, and alcohol consumption. β: beta value for linear regression estimate, km: kilometers.

^1^Sample size n = 615

^2^Sample size n = 741

**Supplemental Table 21**. Sensitivity regression analysis stratified by release type (air, land, water) for KHANDLE and STAR cohorts.

| **Distance to lead facility** | **Cohort** | | | | | | | | | |
| --- | --- | --- | --- | --- | --- | --- | --- | --- | --- | --- |
|  | **KHANDLE** | | | | | **STAR** | | | | |
|  | **N (%)** | **Cognitive measure** | | | | **N (%)** | **Cognitive measure** | | | |
|  |  | **Episodic memory** | **Semantic memory** | **Executive function** | **Global cognition** |  | **Episodic memory** | **Semantic memory** | **Executive function** | **Global cognition** |
|  |  | **β (95% CI)^1^** | **β (95% CI)^1^** | **β (95% CI)^1^** | **β (95% CI)^1^** |  | **β (95% CI)^1^** | **β (95% CI)^1^** | **β (95% CI)^1^** | **β (95% CI)^1^** |
| **Air release > 100 lbs continuous (per 5 km)^2^** |  |  |  |  |  | - | - | - | - | - |
| **No** | 1,607 (98.1%) | - | - | - | - | - | - | - | - | - |
| **Yes** | 31 (1.9%) | 1.2 (0.13, 2.2) | -1.6 (-2.6, -0.64) | -1.9 (-3.0, -0.85) | -0.79 (-1.6, 0.05) | - | - | - | - | - |
| **Air release > 0 continuous (per 5 km)** |  |  |  |  |  |  |  |  |  |  |
| **No** | 843 (51.5%) | - | - | - | - | 363 (49.0%) | - | - | - | - |
| **Yes** | 795 (48.5%) | -0.01 (-0.07, 0.04) | -0.03 (-0.08, 0.02) | -0.03 (-0.08, 0.01) | -0.03 (-0.06, 0.01) | 378 (51.0%) | -0.02 (-0.23, 0.19) | -0.23 (-0.43, -0.03) | -0.10 (-0.31, 0.10) | -0.12 (-0.27, 0.04) |
| **Air release within 1.5 km** |  |  |  |  |  |  |  |  |  |  |
| **No** | 1609 (98.2%) | - | - | - | - | 705 (95.1%) | - | - | - | - |
| **Yes** | 29 (1.8%) | -0.03 (-0.35, 0.28) | -0.01 (-0.29, 0.27) | -0.12 (-0.42, 0.17) | -0.06 (-0.28, 0.17) | 36 (4.9%) | -0.02 (-0.31, 0.27) | -0.07 (-0.37, 0.23) | -0.24 (-0.52, 0.04) | -0.11 (-0.34, 0.11) |
| **Air release within 3 km** |  |  |  |  |  |  |  |  |  |  |
| **No** | 1491 (91.0%) | - | - | - | - | 583 (78.7%) | - | - | - | - |
| **Yes** | 147 (9.0%) | -0.12 (-0.27, 0.03) | -0.13 (-0.26, 0.003) | -0.08 (-0.22, 0.06) | -0.11 (-0.22, -0.004) | 158 (21.3%) | 0.002 (-0.16, 0.16) | -0.07 (-0.23, 0.10) | -0.16 (-0.32, -0.01) | -0.08 (-0.20, 0.04) |
| **Air release within 5 km** |  |  |  |  |  |  |  |  |  |  |
| **No** | 1286 (78.5%) | - | - | - | - | 438 (59.1%) | - | - | - | - |
| **Yes** | 352 (21.5%) | -0.13 (-0.24, -0.03) | -0.13 (-0.23, -0.04) | -0.09 (-0.19, 0.004) | -0.12 (-0.20, -0.04) | 303 (40.9%) | 0.02 (-0.11, 0.15) | -0.06 (-0.19, 0.08) | -0.09 (-0.22, 0.03) | -0.04 (-0.14, 0.06) |
| **Water release within 1.5 km** |  |  |  |  |  |  |  |  |  |  |
| **No** | 1629 (99.5%) | - | - | - | - | 731 (98.7%) | - | - | - | - |
| **Yes** | 9 (0.5%) | -0.04 (-0.60, 0.52) | -0.02 (-0.53, 0.48) | -0.36 (-0.89, 0.16) | - 0.14 (-0.55, 0.26) | 10 (1.3%) | -0.04 (-0.57, 0.50) | -0.22 (-0.76, 0.33) | -0.19 (-0.70, 0.33) | -0.15 (-0.56, 0.26) |
| **Water release within 3 km** |  |  |  |  |  |  |  |  |  |  |
| **No** | 1595 (97.4%) | - | - | - | - | 699 (94.3%) | - | - | - | - |
| **Yes** | 43 (2.6%) | -0.22 (-0.48, 0.04) | -0.27 (-0.50, -0.04) | -0.23 (-0.48, 0.01) | -0.24 (-0.43, -0.06) | 42 (5.7%) | 0.02 (-0.25, 0.29) | 0.05 (-0.23, 0.33) | -0.14 (-0.40, 0.12) | -0.02 (-0.23, 0.18) |
| **Water release within 5 km** |  |  |  |  |  |  |  |  |  |  |
| **No** | 1513 (92.4%) | - | - | - | - | 651 (87.9%) | - | - | - | - |
| **Yes** | 125 (7.6%) | -0.18 (-0.33, -0.02) | -0.17 (-0.31, -0.03) | -0.15 (-0.30, -0.004) | -0.16 (-0.28, -0.05) | 90 (12.1%) | 0.12 (-0.08, 0.31) | 0.05 (-0.15, 0.25) | 0.01 (-0.18, 0.19) | 0.06 (-0.09, 0.21) |
| **Land release within 1.5 km** |  |  |  |  |  |  |  |  |  |  |
| **No** | 1634 (99.8%) | - | - | - | - | 741 (100%) | - | - | - | - |
| **Yes** | 4 (0.2%) | -0.58 (-1.42, 0.26) | -0.08 (-0.83, 0.67) | -0.64 (-1.42, 0.14) | -0.43 (-1.04, 0.17) | 0 (0%) | - | - | - | - |
| **Land release within 3 km** |  |  |  |  |  |  |  |  |  |  |
| **No** | 1628 (99.4%) | - | - | - | - | 740 (99.9%) | - | - | - | - |
| **Yes** | 10 (0.6%) | -0.15 (-0.68, 0.38) | -0.03 (-0.50, 0.45) | -0.28 (-0.77, 0.22) | -0.15 (-0.53, 0.23) | 1 (0.1%) | -0.17 (-1.85, 1.50) | 0.19 (-1.53, 1.90) | 0.79 (-0.81, 2.4) | 0.27 (-1.01, 1.55) |
| **Land release within 5 km** |  |  |  |  |  |  |  |  |  |  |
| **No** | 1617 (98.7%) | - | - | - | - | 738 (99.6%) | - | - | - | - |
| **Yes** | 21 (1.3%) | -0.12 (-0.49, 0.24) | -0.11 (-0.44, 0.22) | -0.29 (-0.64, 0.05) | -0.18 (-0.44, 0.09) | 3 (0.4%) | 0.53 (-0.43, 1.50) | -1.12 (-2.10, -0.13) | 0.35 (-0.58, 1.28) | -0.08 (-0.82, 0.67) |

^1^ Fully adjusted for age, sex, education, census tract income, marital status, race/ethnicity, smoking status, and alcohol consumption. β: beta value for linear regression estimate, km: kilometers. ^2^Exploratory analysis In KHANDLE only, restricted to facilities releasing 100 lbs or more of lead into the air. “Yes” indicates the nearest lead facility releases >0 lbs or >100lbs (for the tested mode of release) and is within the designated distance metric (1.5/3/5 km). “No” indicates either linked facility is not an (air/water/land) release facility or does not fall within the tested distance.

**Supplemental Table 22**. Sensitivity regression analysis using mixed effect model with random effect by census tract for KHANDLE.

| **Distance to lead**  **facility** | **Cognitive measure** | | | | | | | |
| --- | --- | --- | --- | --- | --- | --- | --- | --- |
|  | **Episodic memory** | | **Semantic memory** | | **Executive function** | | **Global cognition** | |
| **Fixed effects** | **β** | **95% CI** | **β** | **95% CI** | **β** | **95% CI** | **β** | **95% CI** |
| Continuous (per 5 km) | -0.03 | (-0.07, -0.001) | -0.02 | (-0.05, 0.01) | 0.01 | (-0.03, 0.04) | -0.02 | (-0.04, 0.01) |
| Within 1.5 km | -0.07 | (-0.29, 0.15) | -0.15 | (-0.35, 0.04) | -0.24 | (-0.44, -0.03) | -0.15 | (-0.32, 0.01) |
| Within 3 km | -0.07 | (-0.18, 0.04) | -0.10 | (-0.20, -0.003) | -0.07 | (-0.17, 0.04) | -0.08 | (-0.16, -0.001) |
| Within 5 km | -0.12 | (-0.22, -0.03) | -0.09 | (-0.17, -0.004) | -0.02 | (-0.11, 0.07) | -0.08 | (-0.14, -0.01) |

^1^ Fully adjusted for age, sex, education, census tract income, marital status, race/ethnicity, smoking status, and alcohol consumption. β: beta value for linear regression estimate, km: kilometers.

**Supplemental Table 23**. Sensitivity regression analysis using mixed effect model with random effect by census tract for STAR.

| **Distance to lead**  **facility** | **Cognitive measure** | | | | | | | |
| --- | --- | --- | --- | --- | --- | --- | --- | --- |
|  | **Episodic memory** | | **Semantic memory** | | **Executive function** | | **Global cognition** | |
| **Fixed effects** | **β** | **95% CI** | **β** | **95% CI** | **β** | **95% CI** | **β** | **95% CI** |
| Continuous (per 5 km) | -0.03 | (-0.15, 0.09) | -0.08 | (-0.20, 0.05) | -0.03 | (-0.14, 0.08) | -0.04 | (-0.13, 0.05) |
| Within 1.5 km | -0.13 | (-0.33, 0.07) | -0.12 | (-0.33, 0.09) | -0.05 | (-0.24, 0.14) | -0.09 | (-0.24, 0.06) |
| Within 3 km | -0.06 | (-0.19, 0.08) | -0.13 | (-0.27, 0.02) | -0.10 | (-0.23, 0.03) | -0.09 | (-0.19, 0.01) |
| Within 5 km | 0.02 | (-0.16, 0.21) | -0.20 | (-0.40, -0.003) | 0.08 | (-0.10, 0.26) | -0.03 | (-0.18, 0.11) |

^1^ Fully adjusted for age, sex, education, census tract income, marital status, race/ethnicity, smoking status, and alcohol consumption. β: beta value for linear regression estimate, km: kilometers.

**Supplemental Table 24.** Extended linear regression analysis adjusting for annual PM_2.5_ (μg/m^3^) for associations between residential distance to lead releasing facility and baseline cognition in the Kaiser Healthy Aging and Diverse Life Experiences Study (KHANDLE) analytic sample (n=1,638). Cognitive measures are z-score standardized.

| **Distance to lead**  **facility** | **Cognitive measure** | | | | | | | | | | | |
| --- | --- | --- | --- | --- | --- | --- | --- | --- | --- | --- | --- | --- |
|  | **Episodic memory** | | | **Semantic memory** | | | **Executive function** | | | **Global cognition** | | |
|  | **β** | **95% CI** | **P value** | **β** | **95% CI** | **P value** | **β** | **95% CI** | **P value** | **β** | **95% CI** | **P value** |
| **Fully adjusted^1^** |  |  |  |  |  |  |  |  |  |  |  |  |
| Continuous (per 5 km) | -0.06 | (-0.09, -0.03) | <0.001 | -0.03 | (-0.06, 0.001) | 0.06 | 0.003 | (-0.03, 0.03) | 0.82 | -0.03 | (-0.05, -0.005) | 0.01 |
| Within 1.5 km | -0.09 | (-0.32, 0.13) | 0.4 | -0.17 | (-0.36, 0.03) | 0.10 | -0.05 | (-0.23, 0.14) | 0.01 | -0.17 | (-0.33, -0.01) | 0.03 |
| Within 3 km | -0.10 | (-0.21, 0.01) | 0.067 | -0.10 | (-0.19, 0.001) | 0.05 | -0.09 | (-0.22, 0.04) | 0.27 | -0.08 | (-0.16, -0.01) | 0.03 |
| Within 5 km | -0.16 | (-0.25, -0.07) | <0.001 | -0.08 | (-0.16, 0.002) | 0.05 | 0.08 | (-0.10, 0.26) | 0.85 | -0.08 | (-0.15, -0.02) | 0.01 |

^1^Fully adjusted for age, sex, education, census tract income, marital status, race/ethnicity, smoking status, alcohol consumption, and annual pm2.5. Continuous distance interpreted per every 5 km closer a residence is to a lead facility. β: beta value for linear regression estimate, km: kilometers; annual PM_2.5_: 1-calendar-year average of daily PM_2.5 (μg/m3)_ measurements two years prior to cognitive testing.

**Supplemental Table 25.** Extended linear regression analysis adjusting for annual PM_2.5_ (μg/m^3^) for associations between residential distance to lead releasing facility and baseline cognition in the Study of Healthy Aging in African Americans (STAR) analytic sample (n=741). Cognitive measures are z-score standardized.

| **Distance to lead**  **facility** | **Cognitive measure** | | | | | | | | | | | |
| --- | --- | --- | --- | --- | --- | --- | --- | --- | --- | --- | --- | --- |
|  | **Episodic memory** | | | **Semantic memory** | | | **Executive function** | | | **Global cognition** | | |
|  | **β** | **95% CI** | **P value** | **β** | **95% CI** | **P value** | **β** | **95% CI** | **P value** | **β** | **95% CI** | **P value** |
| **Fully adjusted^1^** |  |  |  |  |  |  |  |  |  |  |  |  |
| Continuous (per 5 km) | -0.03 | (-0.15, 0.08) | 0.59 | -0.07 | (-0.19, 0.05) | 0.25 | -0.02 | (-0.13, 0.09) | 0.67 | -0.04 | (-0.13, 0.05) | 0.35 |
| Within 1.5 km | -0.13 | (-0.33, 0.07) | 0.20 | -0.08 | (-0.28, 0.12) | 0.45 | -0.04 | (-0.23, 0.14) | 0.64 | -0.08 | (-0.23, 0.07) | 0.28 |
| Within 3 km | -0.06 | (-0.19, 0.08) | 0.40 | -0.10 | (-0.24, 0.03) | 0.14 | -0.09 | (-0.22, 0.04) | 0.19 | -0.08 | (-0.19, 0.02) | 0.12 |
| Within 5 km | 0.03 | (-0.16, 0.21) | 0.76 | -0.21 | (-0.40, -0.02) | 0.03 | 0.08 | (-0.09, 0.26) | 0.36 | -0.03 | (-0.18, 0.11) | 0.64 |

^1^Fully adjusted for age, sex, education, income, marital status, race/ethnicity, smoking status, alcohol consumption, and annual pm2.5. Continuous distance interpreted per every 5 km closer a residence is to a lead facility. β: beta value for linear regression estimate, km: kilometers; annual PM_2.5_: 1-calendar-year average of daily PM_2.5 (μg/m3)_ measurements two years prior to cognitive testing.
